# Optimizing the quasi-equilibrium state of hot carriers in all-inorganic lead halide perovskite nanocrystals through Mn doping: fundamental dynamics and device perspectives†

**DOI:** 10.1039/d1sc05799e

**Published:** 2022-01-14

**Authors:** Jie Meng, Zhenyun Lan, Weihua Lin, Mingli Liang, Xianshao Zou, Qian Zhao, Huifang Geng, Ivano E. Castelli, Sophie E. Canton, Tönu Pullerits, Kaibo Zheng

**Affiliations:** Department of Chemistry, Technical University of Denmark DK-2800 Kongens Lyngby Denmark kzheng@kemi.dtu.dk kaibo.zheng@chemphys.lu.se; Department of Energy Conversion and Storage, Technical University of Denmark DK-2800 Kongens Lyngby Denmark; Chemical Physics and NanoLund, Lund University Box 124 22100 Lund Sweden; Ultrafast Electron Microscopy Laboratory, The MOE Key Laboratory of Weak-Light Nonlinear Photonics, School of Physics, Nankai University Tianjin 300071 China; European XFEL Holzkoppel 4 22869 Schenefeld Germany

## Abstract

Hot carrier (HC) cooling accounts for the significant energy loss in lead halide perovskite (LHP) solar cells. Here, we study HC relaxation dynamics in Mn-doped LHP CsPbI_3_ nanocrystals (NCs), combining transient absorption spectroscopy and density functional theory (DFT) calculations. We demonstrate that Mn^2+^ doping (1) enlarges the longitudinal optical (LO)–acoustic phonon bandgap, (2) enhances the electron–LO phonon coupling strength, and (3) adds HC relaxation pathways *via* Mn orbitals within the bands. The spectroscopic study shows that the HC cooling process is decelerated after doping under band-edge excitation due to the dominant phonon bandgap enlargement. When the excitation photon energy is larger than the optical bandgap and the Mn^2+^ transition gap, the doping accelerates the cooling rate owing to the dominant effect of enhanced carrier–phonon coupling and relaxation pathways. We demonstrate that such a phenomenon is optimal for the application of hot carrier solar cells. The enhanced electron–LO phonon coupling and accelerated cooling of high-temperature hot carriers efficiently establish a high-temperature thermal quasi-equilibrium where the excessive energy of the hot carriers is transferred to heat the cold carriers. On the other hand, the enlarged phononic band-gap prevents further cooling of such a quasi-equilibrium, which facilitates the energy conversion process. Our results manifest a straightforward methodology to optimize the HC dynamics for hot carrier solar cells by element doping.

## Abstract 2

Mn doping modulates the hot carrier dynamics in all-inorganic lead halide perovskite nanocrystals according to the excitation energy.

## Graphical abstract

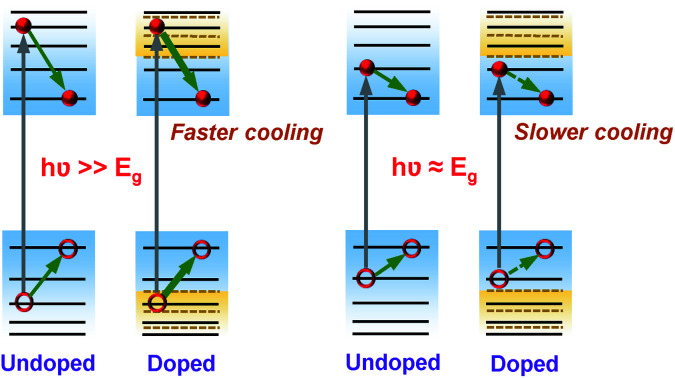

## Introduction

**Scheme 1:**
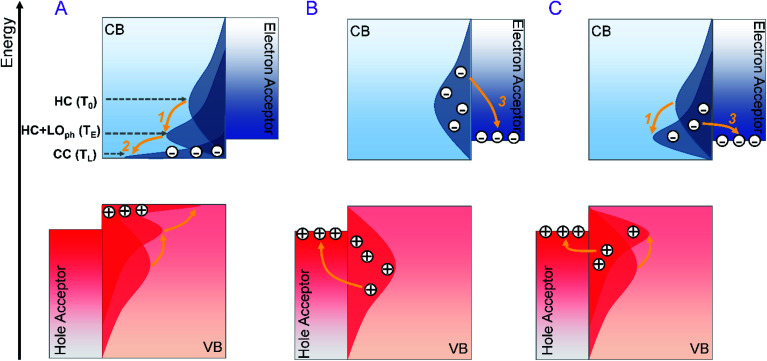
Schematic diagram of HC cooling *versus* HC extraction to charge acceptor electrodes in a HCSC: (a) HC cooling is so efficient that the HC cools from the initial temperature (*T*_0_) to a thermal quasi-equilibrium (*T*_E_) combined with emitted LO phonons (process 1) and finally all reach the cold carrier (CC) state after LO phonon decay (process 2) at a temperature of *T*_L_. (b) HC cooling is extremely slow so that all the HCs are directly injected into acceptors (process 3) without thermalization, where the excess energy is dissipated during the injection. (c) HC undergoes efficient process 1 to establish the thermal equilibrium, and such equilibrium is long-lived to ensure the subsequent HC extraction to the acceptors.

In a single-junction solar cell, the rapid cooling of the hot carrier (HC) excited by photons with energy well above the bandgap is a major energy loss channel responsible for the Shockley–Queisser (SQ) limit.^1^ The hot carrier solar cell (HCSC) is set to tackle this issue by keeping the HC in a sufficiently high energy quasi-equilibrium (*i.e.* with a mean carrier temperature > 500 K) with a subset of the thermal environment directly coupled to the electrons. In this way, the carriers can be extracted at significantly higher energy than the bandgap of the semiconductor, thereby making efficient use of the excess energy of the HC.^2^ An ideal single-junction HCSC can reach a power conversion efficiency as high as 66%.^1^ However, achieving an efficient HC extraction with all the excess energy to be harvested is highly challenging. As illustrated in Scheme 1, previous studies attributed the bottleneck to the suppression of HC extraction by faster HC cooling to the near-band-edge (Scheme 1a), and sought materials with a slow HC cooling rate to ensure the HC injection into electrodes at hot states (Scheme 1b).^3,4^ However, the excess energy of the HC in this case will still be lost in the acceptor electrodes. Utilization of an energy selective electrode (ESC) to screen the energy of the injected hot carrier can be one possible solution. However, it requires a perfect energy alignment between photoactive materials and ESCs, which is difficult to achieve. Therefore, the optimal solution for HCSCs is to modify the HC cooling dynamics of photoactive materials to initiate the efficient establishment of the above-mentioned thermal quasi-equilibrium where the excess energy of the HC is converted to heat the cold carriers. Subsequently, such a quasi-equilibrium state should be long-lived to facilitate the HC extraction (Scheme 1c). In general, the lifetime of the quasi-equilibrium state should be ten times longer than the carrier injection time to acceptors (about 0.l ps) to ensure a sufficient HC harvesting efficiency.^5^

In general, HC cooling in conventional semiconductors mainly involves carrier excitation, carrier–LO-phonon scattering, and LO-phonon decay.^6,7^ The competition between the phonon emission and decay in these processes determines the final HC cooling dynamics.^8^ Among them, the first two steps contribute to the establishment of the thermal quasi-equilibrium, while the last step promotes heat dissipation to dissociate such equilibrium. Therefore, the objectives of HCSC material engineering need to be revised by enhancing carrier–LO-phonon scattering and diminishing LO-phonon decay simultaneously so as to promote a stable thermal quasi-equilibrium.

The emerging lead halide perovskites (LHPs) with promising potential for solar cell applications can be the perfect target materials to evaluate the above strategy. The thermal quasi-equilibrium state is easily formed in LHPs with a long lifetime due to the large polaron screening effect,^9^ hot-phonon effect,^10^ and pronounced acoustical–optical phonon upconversion.^8^ Increasing attention has been focused on the HC dynamics in these materials since the first report of HC cooling in methylammonium lead iodide (MAPbI_3_) polycrystalline thin films.^11,12^ Highly efficient HC extraction (up to ≈83%) could be achieved by an energy-selective electron acceptor layer from a surface-treated MAPbBr_3_ LHP nanocrystal (NC) film.^13^ In formamidinium Sn-based perovskite FASnI_3_, a long HC cooling lifetime of up to a few ns was reported.^14^ In particular, the transport of a persistent HC over long distances (up to ≈600 nm)^3^ makes LHPs greatly promising for HCSCs.

In parallel, transition metal doping has been widely explored to impart novel optical, magnetic, and electronic properties to LHPs.^15^ A representative study in this regard is the Mn doping of LHP materials (*e.g.*, CsPbCl_3_).^16–18^ Such doping can generate long-lived sensitized dopant luminescence and create a magnetically-coupled exciton state.^19^ The unique Mn^2+^ triplet emission in doped LHPs is derived from the Mn^2+^ d–d transition (^4^T_1_ → ^6^A_1_) after energy transfer or charge transfer from the excited state of the host LHPs.^20^ In addition, we have previously demonstrated that partial replacement of Pb^2+^ by Mn^2+^ also causes local structural distortions and defect formation due to the difference in the cation radius between Mn^2+^ and Pb^2+^.^21^ It should be noted that the HC dynamic processes are determined by both electronic and phononic structures of the material. For instance, an enlarged phononic bandgap between optical phonon and acoustic phonon branches can efficiently suppress the channel for LO phonon decay, while a small LO phonon energy requires more phonons to be emitted for a given energy loss of a HC.^22^ Since both electronic states and local structures are modified by Mn^2+^ doping in LHPs, we thereby expect that it can be a robust tool to also modulate HC cooling dynamics towards enhanced carrier–LO-phonon scattering and diminished LO-phonon decay as mentioned above.

**Fig. 1:**
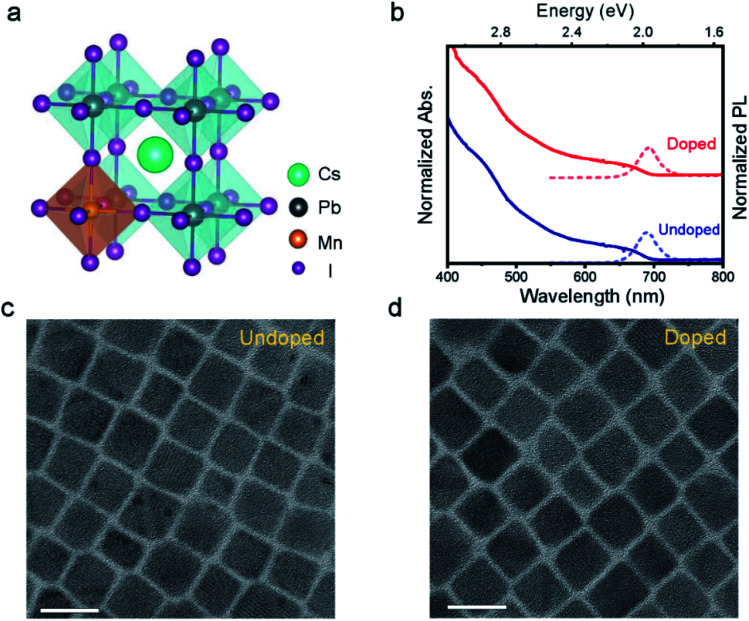
(a) Schematic structure of Mn-doped CsPbI_3_ NCs. The doping concentration is 5%. (b) The absorption (solid line) and PL spectra (dashed line) of undoped (bottom, black line) and Mn-doped (top, red line) CsPbI_3_ NCs. TEM image of (c) undoped and (d) Mn-doped CsPbI_3_ NCs. Examination of the TEM pictures shows that the Mn ions are not located at the surface of the NCs.

In this work, we investigated the HC cooling dynamics in Mn-doped CsPbI_3_ nanocrystals (NCs) using transient absorption (TA) spectroscopy combined with theoretical and experimental characterization of the material structures. Compared to CsPbCl_3_ and CsPbBr_3_, CsPbI_3_ has a lower optical bandgap so that the Mn d orbitals are located within the conduction band (CB) and valence band (VB), and thereby contribute to the cooling pathways. We found that Mn doping modifies the HC cooling rate, but the trend is excitation energy-dependent. We have observed an acceleration in HC cooling at high excitation energy, but a deceleration in HC cooling at near-band-edge excitation energy. The temperature-dependent photoluminescence (PL) measurements and density functional theory (DFT) calculations confirmed the addition of in-band states and enhancement in the carrier–LO phonon coupling by Mn doping. On the other hand, Mn doping enlarged the phonon bandgap between the optical modes and the acoustic modes. We believe that the trade-off among these factors is the main reason for the excitation energy-dependent HC cooling dynamics in Mn-doped LHP NCs. The enhanced carrier–LO phonon coupling and enlarged phonon bandgap comply well with the optimal feature of HCSC materials described in Fig. 1c. This work demonstrates that HC cooling dynamics can be optimized by element doping that tailors the electronic as well as the lattice structure of the materials for the application of HCSCs.

## Results and discussion

### Sample characterization

Both pristine CsPbI_3_ and Mn-doped CsPbI_3_ NCs were synthesized by a reported hot-injection method (details are given in the Experimental section).^23^Fig. 1a schematically shows how Mn metal ions can be incorporated into perovskite lattices with homovalent B-site substitution (ionic radii: Pb^2+^: 133 pm and Mn^2+^: 97 pm).^19^ The transmission electron microscopy (TEM) images show that doped NCs retain the cubic morphology of undoped NCs with an average particle size of around 17 nm (Fig. 1c, d and S1†). Subsequently, the absorption and emission spectra were measured (Fig. 1b). The absorption band-edge of all NCs is ∼680 nm, corresponding to an optical bandgap (*E*_g_) of ∼1.8 eV. The introduction of Mn does not influence the absorption edge of the CsPbI_3_ NCs. In addition, no dopant-related absorption/emission bands can be observed since the Mn d orbitals are not located within the bandgap of CsPbI_3_.^24^

**Fig. 2:**
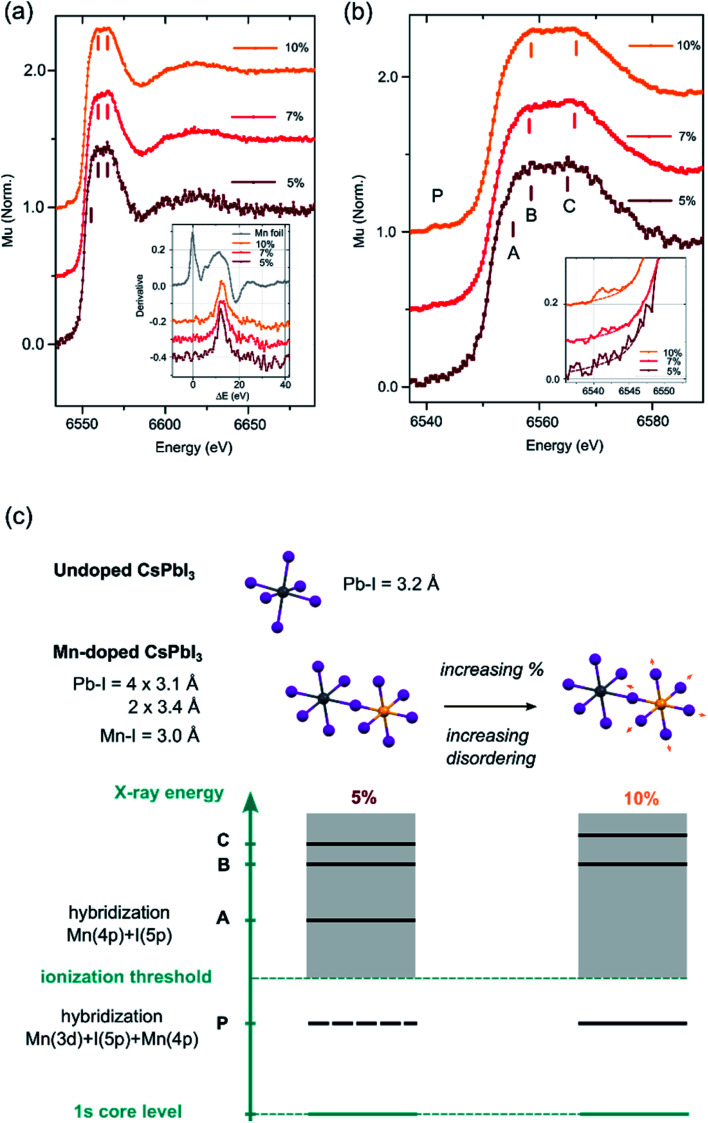
(a) XANES spectra of Mn-doped NCs with various doping concentrations. The inset shows their derivatives along with the one of Mn foil, (b) magnified near edge XANES spectra at the Mn–K edge of Mn-doped NCs with various doping concentrations. The inset shows a zoom of the pre-edge region (the dotted lines are added as guides) and (c) proposed structural motif representing the octahedra in undoped and Mn-doped NCs and schematics of quasi-molecular energy levels used for interpreting the XANES spectral features.

X-ray absorption spectroscopy (XAS) measurements were then conducted at the Mn K-edge to confirm the Mn^2+^ doping into the perovskite lattice and to further characterize the local bonding environment around the Mn^2+^ ions (for experimental details, see the ESI†). Fig. 2a displays the XAS profiles of the samples containing 5% (brown), 7% (red) and 10% Mn (orange). The parameter Δ*E* is defined as the difference between the incident photon energy and the threshold value of Mn^0^.^25^ The inset shows the first derivative of the XANES profiles as a function of Δ*E* for the 3 samples (orange, red and brown lines) and reference Mn foil (grey line). The energy position of the first inflection point correlates with the effective oxidation state of the absorbing atom. For the 3 samples, it falls between the values observed in Mn-oxides of +2, +3 and +4 valencies.^26–28^ This observation confirms that Mn is not present as metallic Mn^0^ clusters, but as Mn^2+^ ions incorporated into the perovskite lattice. The aggregation at the surface of the NCs can be ruled out based on the homogeneous atomic contrast in HR-TEM images. Fig. 2b shows a zoom of the X-ray absorption near-edge (XANES) region. The XANES profile consists of a weak pre-edge feature P centered around 6541 eV and a strong white line at 6560 eV. Forbidden in ideal octahedral symmetry, the pre-edge feature arises from the transition of a Mn 1s electron to the unoccupied Mn 3d levels hybridized with the ligand 4p and Mn 4p levels as a consequence of symmetry lowering and distortions.^29,30^ This feature is observed for 7% and 10% samples (inset of Fig. 2b); but not for 5% due to the lower S/N ratio. The white line is less intense than in materials where Mn^2+^ is coordinated by low-Z elements (*i.e.* Mn oxides).^25^ The white line of the Mn-doped NCs is attributed to the transition of a Mn 1s electron to the molecular levels built from unoccupied 4p levels. In Mn-containing materials (particularly with perovskite structures), the fine structure of the white line is affected by the Mn 3d–4p exchange interaction,^31^ the hybridization of Mn 4p by the ligand orbitals^32^ and the balance between Mn 3d occupation and the Madelung constant^33,34^ and the degree of disorder.^35^ At a low doping concentration (*i.e.*, 5%), the white line presents a distinct shoulder A which can be ascribed to the hybridization of Mn 4p, along with a clear double peak structure (B and C). As the doping concentration increases (7% and 10%), the intensity of P increases, A disappears and the double structure B–C broadens (see Fig. S0†). All these spectral features signal a more pronounced disordering around the Mn atoms, which might be accompanied by a slight elongation of the average Mn–I bond (Fig. 2c). Further work is required to explore the systematic variations of the XANES features in Mn-doped lead-based perovskites.

**Fig. 3:**
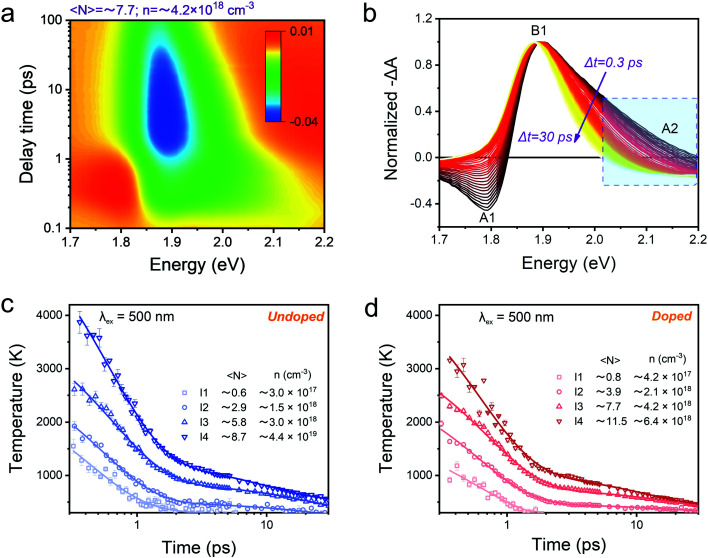
(a) Pseudocolor representation of TA spectra of Mn-doped CsPbI_3_ NCs for 500 nm (2.48 eV) excitation at high excitation intensity with 〈*N*〉 ≈ 7.7 (corresponding to *n* ≈ 4.2 × 10^18^ cm^−3^). (b) Normalized TA spectra of Mn-doped CsPbI_3_ NCs for 2.48 eV excitation at high excitation intensity with 〈*N*〉 ≈ 7.7 (corresponding to *n* ≈ 4.2 × 10^18^ cm^−3^) with a time delay from 0.3 ps to 30 ps. The higher energy tails (between 2.02 and 2.2 eV marked by a dashed rectangle) are globally fitted to a Boltzmann distribution from which the carrier temperature is extracted. Carrier temperature decay kinetics with 2.48 eV excitation at four different excitation intensities for (c) undoped and (d) Mn-doped CsPbI_3_ NCs. The solid lines are the multi-exponential fits, and fitting parameters can be seen in the ESI.†

Next, we studied the HC cooling dynamics of the Mn-doped NCs mainly *via* transient absorption spectroscopy (TA) under various excitation energies and intensities. Fig. 3a shows a pseudo-color TA plot of Mn-doped CsPbI_3_ NCs excited at 500 nm and high excitation intensity with an average number of excitons per NCs 〈*N〉* ≈ 7.7 corresponding to an initial average carrier density *n* ≈ 4.2 × 1^18^ cm^−3^ (for details of pump fluence and absorption coefficient calculations, see Fig. S2, S3 and Table S1†). All the other pseudo-color TA plots with different excitation energies and intensities for undoped and doped NCs are summarized in Fig. S4–S9.† In general, the TA plot shows three distinct spectral features (see Fig. 3b): (1) the ground state bleach (GB) band centered at around 1.9 eV that arises from the near-band-edge state filling (B1); (2) the excited state absorption (ESA) above the bandgap (larger than 1.9 eV) that later evolved into a GB signature (A2); (3) toward the lower energy, the asymmetric derivative is the probe-induced Stark effect (A1).^6^ In addition, we can observe a red shift of the GB peak position with the delay time (Fig. 3a), which becomes more pronounced at high excitation fluences (Fig. S4–S9†). Such a GB position shift can be attributed to two effects: the Moss–Burstein effect and the bandgap renormalization at short timescales (before 2 ps).^36^ The Moss–Burstein effect refers to the increase of the effective bandgap of a semiconductor when the lower energy states in the CB or VB have been populated or blocked.^37^ On the other hand, bandgap renormalization is induced by the screening of Coulomb repulsion leading to a decrease in the electronic bandgap of semiconductors.^38^ Those two effects should compensate each other near the band-edge of the LHPs, resulting in a slight shift in the observed optical band-edge.

Fig. 3b shows the normalized TA spectra −Δ*A* of Mn-doped CsPbI_3_ NCs with a delay time between 0.3 and 3 ps extracted from the TA plot in Fig. 3a. In Mn-doped CsPbI_3_ NCs, after high-energy photon excitation, the excited carriers first undergo carrier–carrier scattering within 100 fs, a process which is called carrier thermalization.^39^ The HC then reaches a Fermi–Dirac distribution with a carrier temperature *T*_c_ larger than the lattice temperature *T*_L_. Following carrier thermalization, the HC equilibrates with the lattice mainly through an inelastic carrier–phonon interaction, known as the “cooling” process. To ensure that the HCs have redistributed their energies and reached a quasi-temperature as the Boltzmann distribution, we analyzed the HC cooling dynamics after a delay of 0.3 ps, when the initial thermalization process should be finished. In order to extract the HC temperature, we fitted the high energy tail of the TA spectra (*i.e.*, between 2.02 eV and 2.20 eV) using the Maxwell–Boltzmann distribution function:^36^1
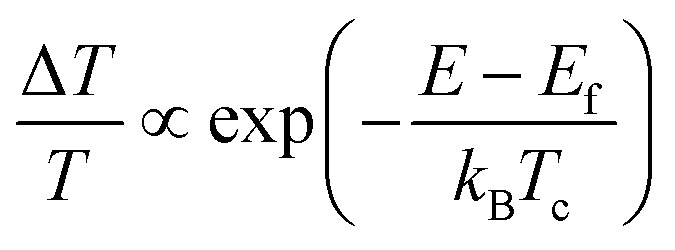
where Δ*T* is the TA signal in the region of interest, *E*_f_ is the quasi-Fermi energy, *k*_B_ is the Boltzmann constant, and *T*_c_ is the carrier temperature. Since electrons/holes show similar effective masses based on the calculations (pure CsPbI_3_: *m*_e_ = 0.10*m*_0_, *m*_h_ = 0.15*m*_0_; Mn-doped CsPbI_3_: *m*_e_ = 0.13*m*_0_, *m*_h_ = 0.19*m*_0_), we expect comparable contributions from hot electrons and hot holes to the extracted HC temperatures. Fig. 3c and d shows the fitted HC cooling dynamics of undoped and doped samples with increasing carrier densities (or pump fluence), respectively The HC cooling rates become lower with increasing carrier densities in both doped and undoped samples. The HC cooling decay can be well-fitted by bi-exponential functions with a fast component and a slow component. The fast component should be attributed to the emission of the LO phonons through carrier–phonon interactions, and the slow component should be due to the reduction of the energy loss rate by reduced decay of LO phonons.^40,41^ We then used the average time to compare the cooling rates of undoped and doped NCs. At low excitation density below 10^18^ cm^−3^, the average decay times are 1.8 ps and 0.6 ps for undoped and doped NCs, respectively. When excitation density is higher than 10^18^ cm^−3^, the average decay times for undoped and doped NCs reach 20.2 ps and 17.9 ps, respectively (Tables S4 and S5†). Notably, the same trend occurs in all the samples with various excitation photon energies (Fig. S10–S17 and Tables S2–S7†). The enhanced hot-phonon bottleneck can explain this general carrier density dependence of HC cooling dynamics, which is widely observed in semiconductors and LHPs.^6,10,39,42,43^ It is mainly induced by the presence of a non-equilibrium LO-phonon population in the phonon pool that reduces the net LO-phonon emission and enhances the cold carrier reheating, which consequently slows down the cooling process.^6,39,44^

### Excitation energy-dependent role of Mn doping in HC dynamics

**Fig. 4:**
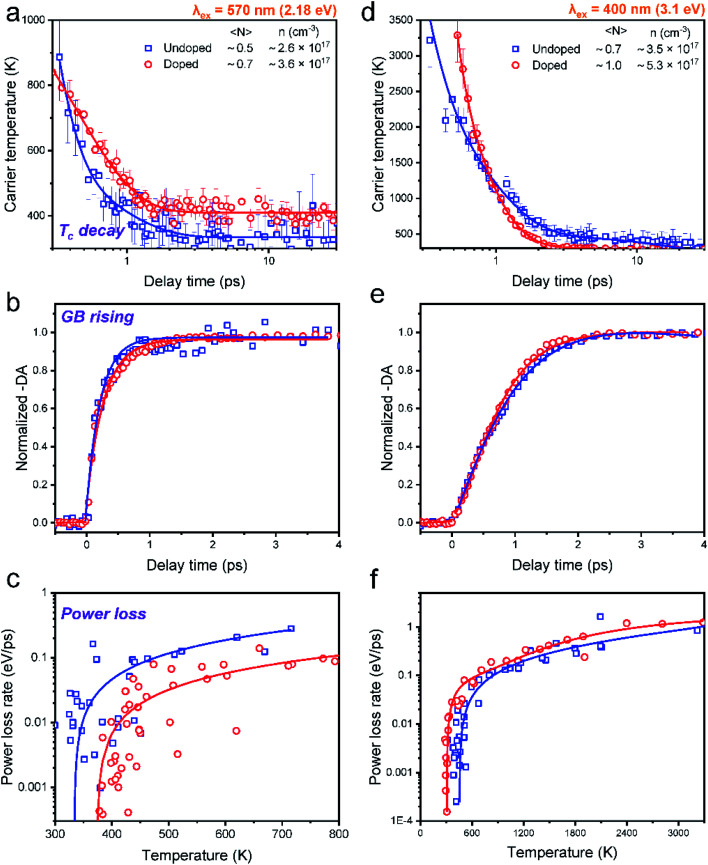
Time-dependent carrier temperature for undoped and Mn-doped CsPbI_3_ NCs at low excitation density (〈*N*〉 < 1) with (a) 2.18 eV and (b) 3.1 eV excitation energies. Normalized GB dynamics probed at the band-edge for undoped and Mn-doped CsPbI_3_ NCs at low excitation intensity with (c) 2.18 eV and (d) 3.1 eV excitation energies. Power loss as a function of the carrier temperature of Mn-doped CsPbI_3_ NCs and corresponding exponential fitting at low excitation intensity with (e) 2.18 eV and (f) 3.1 eV excitation energies.

In order to elucidate the influence of Mn doping on HC cooling, we mainly compared the cooling dynamics between undoped and Mn-doped CsPbI_3_ NCs excited at different excitation energies at very low excitation intensity (〈*N*〉 ≤ 1) (Fig. 4). Although such excitation density is still higher than real sunlight radiation conditions, the low excitation density per NC in our system can guarantee that the many-body effects influencing HC cooling, such as Auger reheating, are negligible.^13^

We can fit all the HC cooling decay processes by multiple exponential components as summarized in Table 1. When the samples are excited at 2.18 eV with the phonon energy close to the bandgap energy, the cooling decay of the undoped NCs can be fitted with a fast component (0.1 ps) and a slow component (0.7 ps), delivering an average lifetime of 0.3 ps (Fig. 4a). Upon Mn doping, only one exponential component can be fitted with a longer lifetime of 0.4 ps (also evidenced by the log plot of the kinetics in Fig. S22†). This manifests that HC cooling in doped NCs becomes slower with the excitation energy near the band-edge position (Fig. 4a). It should be noted that due to the limited spectral region which can be utilized for the *T*_c_ fitting under 2.18 eV excitation conditions where the GB tail is close to the scattering of the pump pulse, the fitted minimum *T*_c_ is a bit above 300 K. Nevertheless, we believe the trend of the cooling dynamics should not be influenced as the fitting parameters are kept constant for data points from each time delay.

**Table 1:** Fit parameters for carrier temperature decay kinetics of Mn-doped CsPbI_3_ NCs with 3.1 eV and 2.18 eV excitations. The unit of time *t* is ps. The doped sample excited at 2.18 eV can be well fitted by a single exponential function, which can be justified from the ln *T vs. t* plot in Fig. S22

Exc. Wavelength		*A*_1_	*t*_1_	*A*_2_	*t*_2_	*t*_ave_
3.1 eV	Undoped	0.85	0.2	0.15	0.9	0.5
	Doped	0.97	0.1	0.03	0.5	0.2
2.18 eV	Undoped	0.95	0.1	0.05	0.7	0.3
	Doped	1	0.4	—	—	0.4

When the sample is excited at 3.1 eV, the kinetics for the undoped NCs can be fitted by a fast component (0.2 ps) and a slow component (0.9 ps), giving an average lifetime of 0.5 ps. Upon Mn doping, the average lifetime becomes much faster (0.2 ps) (Fig. 4b). The same trend can also be seen in the excitation energy of 2.48 eV (Fig. S21†) (for detailed fitting parameters, see Table S11†). The above excitation energy-dependent comparison can be further confirmed by the increasing time of TA kinetics at the maximum GB position, which monitors the population of the band-edge states (Fig. 4c and d).

In general, HC cooling dynamics in semiconductors are related to the coupling between various thermal pools. Here the above analysis monitors the temperature of the electronic pool, which is coupled to the pool of LO phonons.^13^ Owing to the anharmonicity of the vibrations, the LO phonon pool transfers energy efficiently to the acoustic phonon pool followed by the energy dissipation to the solvent environment.^45^ In order to identify the dominant steps of HC relaxation at various time delays, we have extracted the electronic energy power loss as a function of carrier temperature by using the conventional model of hot electron relaxation *via* optical-phonon emissions in Fig. 4e and f.^13^ The energy loss rate (*P*) of the carriers can be derived from the equation 
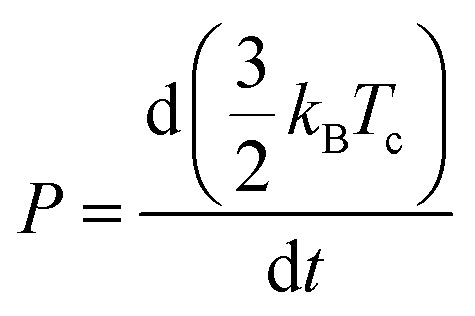
 where *T*_c_ refers to the carrier temperature. The power loss plots in Fig. 4e and f further confirm that HC cooling in doped NCs is slower under 2.18 eV excitation and faster under 3.1 eV excitation compared with doped NCs. In addition, two distinct slopes can be observed in the plot corresponding to two power loss regimes. As shown in Fig. 4f, for the undoped NCs excited at 3.1 eV, the power loss rate slowly decreases from 1 to 0.2 eV ps^−1^, until *T*_c_ reaches ≈700 K. Subsequently, as the HC temperature approaches the lattice temperature, *P* drops rapidly. We defined the temperature at the transition point between these two regions as transition temperature (*T*_r_). When the samples are excited at 2.18 eV, the *T*_r_ for the undoped and doped NCs is about 410 K and 450 K, respectively (Fig. 4e). At 3.1 eV excitation, the *T*_r_ for the undoped and doped NCs is about 780 K and 580 K (Fig. 4f), respectively. Mn doping leads to a lower *T*_r_ under 3.1 eV excitation and a higher *T*_r_ under 2.18 eV excitation. The two distinct regions of HC power loss are mainly dependent on the electronic and phononic structures of the materials. Notably, at a low carrier density (∼10^−17^ cm^−3^), the power loss of the HC during the cooling process is dominated by the scattering between carriers and LO phonons. The initial rapid HC cooling (*i.e.*, the higher power loss rate) with the *T*_c_ above the *T*_r_ is due to the efficient LO phonon emission through the dominant Fröhlich interaction that dissipates the excess energy of the HC. These LO phonons decay into acoustic phonons until the *T*_c_ cools to the lattice temperature. The subsequent slower cooling of the HC closer to the near-band-edges (*i.e.*, around 300–600 K in Fig. 4e) is determined by the thermal equilibration between LO phonons and acoustic phonons.^13^ In this scenario, the *T*_r_ between the two power loss regions qualitatively reflects the impact of the hot-phonon bottleneck, which is determined by the population dynamics of non-equilibrium LO phonons in the phonon pools. At high excitation energy, the *T*_r_ values of undoped and doped NCs are generally increased compared to low excitation energy as shown in Fig. 4e and f. This can be explained as more LO phonons are emitted due to the higher excess energy of the excited HC. On the other hand, the change of the *T*_r_ after Mn doping in NCs indicates modified phonon generation and decay dynamics.

**Fig. 5:**
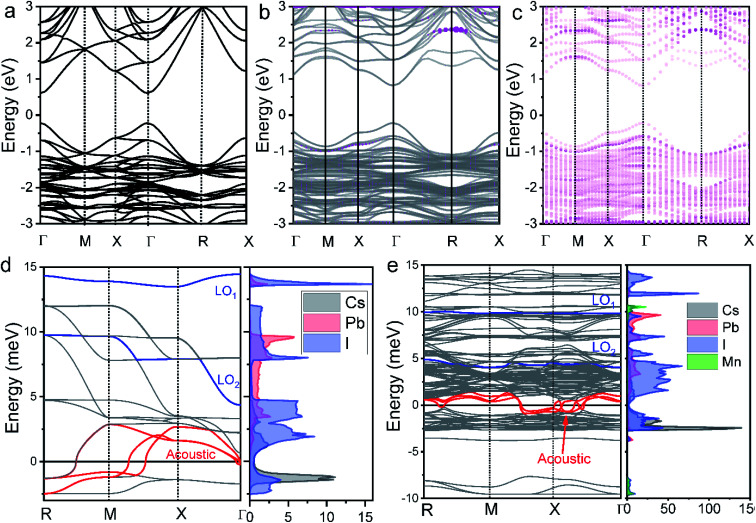
Electronic band structure of (a) CsPbI_3_ and (b) Mn-doped CsPbI_3_ including Mn orbitals shown by pink color. (c) The contribution of Mn d orbitals in the electronic band structure of Mn-doped CsPbI_3_. Phonon energy as a function of phonon momentum and density of states (DOS) of (d) undoped CsPbI_3_ and (e) Mn-doped CsPbI_3_. The size of these circles is proportional to the contribution of the corresponding orbitals. The two blue lines indicate the LO phonon mode. The red lines indicate the acoustic phonon mode.

In the following, we interpret the excitation energy-dependent role of Mn doping in the HC cooling dynamics observed in Fig. 4 from the intrinsic electronic/phononic structure of the doped NCs. We first revealed the electronic band structure of the samples *via* DFT calculations, as shown in Fig. 5a and b. The effective electron mass and hole mass of Mn-doped CsPbI_3_ (*m*_e_ = 0.13*m*_0_ and *m*_h_ = 0.19*m*_0_) are larger than those of pure CsPbI_3_ (*m*_e_ = 0.10*m*_0_ and *m*_h_ = 0.15*m*_0_). This is because of the perturbation in the periodicity of the Pb 6p orbitals after Mn doping which leads to the reduction of both VBM and CBM dispersion. This indicates a more localized electron and hole state in the doped NCs.^46^ In the absence of the hot-phonon effect at low carrier concentrations, the energy loss rate of the HC *via* carrier–LO phonon interactions is predominantly affected by the effective mass as illustrated in eqn (2).^6^2
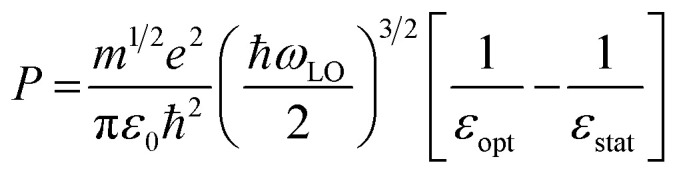
where *ε*_Opt_ and *ε*_Stat_ are the optical and static dielectric constants. Therefore, the heavier carrier effective mass in doped NCs should induce an intrinsically faster HC relaxation than in undoped NCs.

Secondly, we calculated the density of states (DOS) of pure CsPbI_3_ NCs and the Mn-doped levels shown in Fig. 5a–c. For the undoped NCs, the pathways of HC cooling are mediated by states built from Pb and I orbitals. The doping adds Mn orbitals into the electronic structures of the NCs as shown in Fig. 5c. According to the previous theoretical calculations, the first excitation at every symmetry point shows high optical strength.^47,48^ As shown in Fig. 5b, when the doped samples are excited at high energy, (*e.g.*, 3.1 eV) more channels are available for electronic relaxation of the HC. When the doped samples are excited at near band-edge energies, the excited electrons/holes possess energy lower than the majority of the Mn orbitals. Therefore, we believe that the participation of the Mn orbital in HC cooling should be negligible.

Following the discussion of the effect of electronic structure, we now examine the role of carrier–phonon coupling. For most inorganic semiconductors, coupling or scattering between charge carriers and phonons is a functional dependence of the PL linewidth *Γ*(*T*) on temperature.^49^ In order to evaluate such coupling, temperature-dependent PL spectra were acquired. They are shown in Fig. S18 and S19.† We calculated the electron–phonon coupling strength from the FWHMs of temperature-dependent PL spectra using the following model:^50^

3 *Γ*(*T*) = *Γ*_0_ + *Γ*_ac_ + *Γ*_LO_ + *Γ*_imp_ = *Γ*_0_ + *γ*_ac_*T* + *γ*_LO_*N*_LO_(*T*) + *γ*_imp_e^−*E*_b_/*k*_B_*T*^

where *Γ*_0_ is a temperature-independent inhomogeneous broadening that arises from scattering due to disorder and imperfections. *Γ*_ac_ is the contribution from acoustic-phonon scattering and *γ*_ac_ is the corresponding phonon-coupling strength. *Γ*_LO_ corresponds to the homogeneous broadening that results from LO-phonon scattering with a coupling strength *γ*_LO_. In *N*_LO_ (*T*) = 1/(e^*E*_LO_/*k*_B_*T*^ − 1), *E*_LO_ is an energy representative of the frequency for the weakly dispersive LO phonon branch, and *k*_B_ is the Boltzmann constant. *Γ*_imp_ is the inhomogeneous broadening due to the ionized impurities. *Γ*_ac_ and *Γ*_imp_ do not contribute much to the temperature dependence at higher temperatures (>100 K) so that they can be treated as constant and merged into *Γ*_0_. Hence, we model the linewidth broadening of the samples using eqn (4) (Fig. S19†), and the linewidth parameters are shown in Table 2.

4 *Γ*(*T*) = *Γ*_0_ + *γ*_LO_*N*_LO_(*T*)

**Table 2:** Linewidth parameters and calculated *E*_LO_ extracted from Fig. S18 and S19

Samples	*Γ*_0_/meV	*γ*_LO_/meV	*E*_LO_/meV (fitting)	*E*_LO-min_/meV (calculated)	*E*_LO-ma*x*_/meV (calculated)
Undoped	51.25	74.8	23.79	4.3	14.0
Doped	52.87	126.5	30.45	4.1	9.9

Specifically, the LO phonon term in eqn (4) accounts for the Fröhlich interaction between LO phonons and carriers. The difference between fitted and calculated *E*_LO_ is within the experimental error range. The fitted *γ*_LO_ for undoped NCs (74.8 meV) is 1.7 times smaller than that of doped NCs (126.5 meV). This demonstrates the strengthened electron–LO phonon coupling by Mn doping.

The last critical factor for determining HC cooling dynamics is the phonon band structures that dominate the pathways of LO-phonon decay and energy transfer to the acoustic phonons. We performed first-principles calculations of phonon dispersion spectra to uncover the possible phonon decay dynamics (for detailed calculations, see the ESI†). The projected DOS on each atom is also given in Fig. 5d and e to show the detailed contributions from each atom. As well accepted, the most efficient pathway for LO phonon decay to acoustic phonons is the Klemens channel, where one optical phonon decays into two acoustic phonons with symmetric momentum.^51^ However, the Klemens decay requires that the phononic bandgap between LO and acoustic phonons (ℏ*ω*_LO-min_ − ℏ*ω*_LA-ma*x*_) is lower than the maximum ℏ*ω*_LA_ (ℏ*ω*_LA-ma*x*_) energy (*i.e.*, ℏ*ω*_LO-min_ − ℏ*ω*_LA-ma*x*_ < ℏ*ω*_LA-ma*x*_).^52^ Otherwise, the large phononic bandgap hinders the LO phonon decay and hence leads to the formation of a non-equilibrium phonon population where a LO hot-phonon bottleneck arises. In the undoped CsPbI_3_ NCs, the majority of the LO vibrational modes are the Pb–I stretching vibrations with frequencies at around 4.4 meV (ℏ*ω*_LO2-min_) and 13.5 meV (ℏ*ω*_LO1-min_), while the maximum acoustic LA phonon frequency lies around 2.9 meV (ℏ*ω*_LA-ma*x*_) (Fig. 4d). The gap between LO and LA (ℏ*ω*_LO-min_ − ℏ*ω*_LA-ma*x*_) is calculated to be about 1.5 meV. The lower LO phonon energy compared with twice the LA phonon energy guarantees an efficient Klemens decay in the undoped NCs. In contrast, the Pb–I stretching vibration (LO) frequencies in the doped NCs are around 4.1 meV (ℏ*ω*_LO2-min_) and 9.9 meV (ℏ*ω*_LO1-min_), while the maximum acoustic phonon frequency LA lies around 1.3 meV. The gap between LO and LA (ℏ*ω*_LO-min_ − ℏ*ω*_LA-ma*x*_) is about 2.8 meV. Such enlargement of the phonon bandgap between LO and acoustic phonons should be induced from the strain due to Mn doping in the local structures.^53^ The separation between the LO and LA phonon branches (ℏ*ω*_LO-min_ > 2ℏ*ω*_LA-ma*x*_) after Mn doping should significantly hinder the Klemens channel, so that the LO phonons can only decay *via* alternative less efficient channels, such as the Ridley channel.^51^ This suggests that more LO phonons will be emitted to cool down the HC with the same excess energy, which inevitably leads to a large non-equilibrium LO phonon population.

**Fig. 6:**
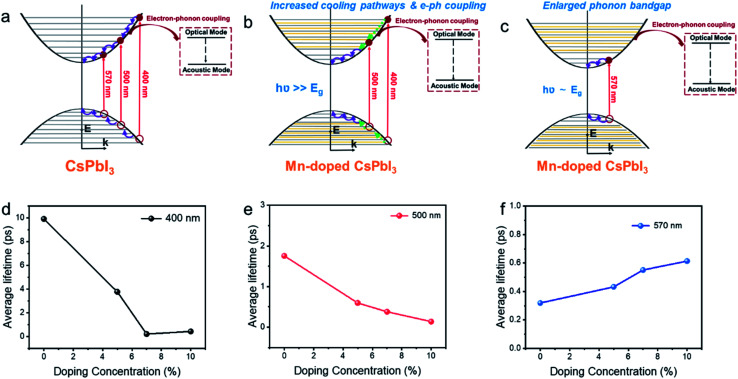
Schematic mechanism diagram of hot carrier cooling for Mn-doped CsPbI_3_ with (a) undoped CsPbI_3_ NCs, (b) Mn-doped CsPbI_3_ with high energy excitation, (c) Mn doped CsPbI_3_ with low energy excitation NCs and average lifetime of hot carrier cooling for Mn-doped CsPbI_3_ with different doping concentrations at (d) 400 nm (3.1 eV), (e) 500 nm (2.48 eV) and (f) 570 (2.18 eV) excitation energies, respectively. The added orange lines in Fig. 5b and c represent Mn dopant orbitals.

We believe that the three factors above, *i.e.* integration of the doping levels, electron–LO phonon coupling, and the phononic band structure, all influence HC cooling dynamics. Therefore, the excitation wavelength dependence of HC cooling dynamics as shown in Fig. 6 should be induced by the competition among all of these factors as summarized in Fig. 6a–c. Under band-edge excitation (*e.g.* excited at 2.18 eV), the influence of additional Mn orbitals within the CB and VB can be neglected as their energies are well above the excited states, as shown in Fig. 5c. We also demonstrated that at high excitation intensity (〈*N*〉 >1), the influence of Mn doping on HC cooling dynamics is significantly diminished (Fig. S23†). Firstly, under a high excitation fluence, the carriers will accumulate towards energy states higher than the Mn dopant orbitals even at low excitation energy (*i.e.* 2.2 eV) due to the so-called Moss–Burstein effect. In this case, Mn dopant orbitals can participate in HC cooling both under band-edge and high energy excitations. Secondly, the pronounced Auger heating effect and the hot phonon bottleneck may overwhelm the influence of intrinsic electronic and phononic structural changes induced by Mn doping. As a result, the HC cooling dynamics between undoped and doped NCs become harder to distinguish (Fig. S4, S5, S8 and S9†). Here the increased phononic bandgap induced by Mn doping is the dominant factor for HC cooling dynamics. As discussed, it hinders the decay of excited LO phonons and promotes the non-equilibrium phonon population, while enhancing the hot-phonon bottleneck. This accounts for the slow HC cooling and high *T*_r_ in power loss for doped NCs as shown in Fig. 6c.

Under high energy excitation (*e.g.* excited at 400 and 2.48 eV), the participation of the Mn orbital in the cooling pathways, together with other factors induced by Mn doping should all play a role in HC cooling dynamics as discussed above (Fig. 6b). The faster cooling rates for doped NCs in this scenario indicate the influence of the Mn orbital participation, and the enhanced e–ph coupling competes with the enlargement of the phonon bandgap to accelerate the cooling process.

Summarizing the role of Mn doping in the HC with various energies, we noticed that such a situation does fit the optimal scenario for the materials applied in HCSCs. The HC at very high energy will undergo strong electron–phonon coupling to be efficiently thermalized to the quasi-equilibrium state integrating the carriers with LO phonons to keep the thermal pool ‘warm’ enough. In this case, the carrier temperature reaches 400 to 600 K reflected by the *T*_r_ values. The phonon bandgap enlargement prevents further heat dissipation when the quasi-equilibrium is established and the HC possesses lower energy.

### Role of the dopant concentration in HC cooling dynamics

The samples with different doping levels are now considered. From the average HC cooling time t_ave_ shown in Fig. 6d–f, we find that the cooling rates become slower with increasing doping concentration under low excitation energies (2.18 eV, Fig. 6f). Since the HC cooling process at low excitation energy is exempt from the Mn orbital participation as discussed above, such a relationship indicates that the combined influence from the change of phononic structures and e–ph coupling evolves monotonously with the doping concentration. According to the XANES characterization above (Fig. 2), the local lattice disordering around the Mn atoms increases monotonously with the doping level increment. Such local distortion should modify the metal-halide vibrational modes, thus enhancing e–ph coupling and accelerating HC cooling in perovskite materials.^54^ On the other hand, local structural distortion should also modify the phononic structure accounting for the enlargement of the phonon bandgap that hinders HC cooling according to the above DFT calculations. Apparently, the influence of phonon bandgap enlargement becomes more dominant at high doping concentrations.

On the other hand, at a high excitation energy of 3.1 eV, the average cooling time first decreases and then increases with the doping concentration. At an excitation energy of 2.48 eV, the average cooling time decreases with the doping concentration. The XANES characterization confirms the monotonously increased disordering of the Mn–I bonds with the doping level, which should weaken the mixing between Mn and I orbitals and affect the available states in the pathway for the cooling of the HC proved by the DFT calculations (Fig. 5c). The above dependences again indicate that Mn orbital pathways (*i.e.* factors that accelerate HC cooling) dominate HC cooling at low doping concentrations. In contrast, the phonon bandgap enlargement (*i.e.* factor that decelerates HC cooling) dominates the cooling dynamics at high doping concentrations.

## Conclusions

In summary, the HC cooling dynamics in Mn-doped CsPbI_3_ NCs have been studied using transient absorption spectroscopy combined with theoretical calculations and local structure characterization. Under a high energy excitation of 3.1 eV, Mn doping promotes fast HC cooling rates. However, Mn doping contributes to slowing the HC cooling rate after excitation at 2.18 eV. From DFT calculations, Mn-doped CsPbI_3_ shows higher effective electron/hole masses in comparison with undoped NCs. Meanwhile, temperature-dependent PL characterization further confirmed that Mn doping also helps to strengthen the electron–phonon coupling. However, a significant energy separation between the optical mode and acoustic modes is observed, implying that Mn doping suppresses the efficient Klemens channel for LO phonon decay. The HC cooling process is therefore the consequence of the competition between all the above factors, which leads to the excitation energy and doping concentration dependence. The enhanced electron–phonon coupling and efficient thermalization of the HC at high energy together with delayed heat dissipation after thermalization with the HC at low energy are optimal for HCSC application. Our results open up a new possibility to optimize HC cooling dynamics in HCSC materials *via* element doping with fine control of both electronic and phononic structures of host materials.

## Author contributions

Jie Meng implemented the sample preparation, characterization, data analysis, and manuscript writing. Zhenyun Lan helped with the calculation with the supervision of Ivano E. Castelli. Weihua Lin helped to measure the transient absorption spectroscopies. Mingli Liang and Xianshao Zou helped to conduct the temperature-dependent PL measurement. Huifang Geng contributed to the TA fitting. Sophie E. Canton contributed to the X-ray experiment and analysis. Tönu Pullerits participated in the data analysis and manuscript revision. Kaibo Zheng is the corresponding author and supervised the whole project.

## Conflicts of interest

The authors declare no competing financial interest.

## Supplementary Material

SC-013-D1SC05799E-s001

## References

[cit1] Ross R. T., Nozik A. J. (1982). J. Appl. Phys..

[cit2] Lim S. S., Giovanni D., Zhang Q., Solanki A., Jamaludin N. F., Lim J. W. M., Mathews N., Mhaisalkar S., Pshenichnikov M. S., Sum T. C. (2019). Sci. Adv..

[cit3] Guo Z., Wan Y., Yang M., Snaider J., Zhu K., Huang L. (2017). Science.

[cit4] Hopper T. R., Gorodetsky A., Frost J. M., Müller C., Lovrincic R., Bakulin A. A. (2018). ACS Energy Lett..

[cit5] Takeda Y., Ito T., Motohiro T., König D., Shrestha S., Conibeer G. (2009). J. Appl. Phys..

[cit6] Fu J., Xu Q., Han G., Wu B., Huan C. H. A., Leek M. L., Sum T. C. (2017). Nat. Commun..

[cit7] Lee S. H., Sim H. S., Lee J., Kim J. M., Shin Y. E. (2006). Mater. Trans..

[cit8] Yang J., Wen X., Xia H., Sheng R., Ma Q., Kim J., Tapping P., Harada T., Kee T. W., Huang F., Cheng Y.-B., Green M., Ho-Baillie A., Huang S., Shrestha S., Patterson R., Conibeer G. (2017). Nat. Commun..

[cit9] Zhu H., Miyata K., Fu Y., Wang J., Joshi P. P., Niesner D., Williams K. W., Jin S., Zhu X. Y. (2016). Science.

[cit10] Yang Y., Ostrowski D. P., France R. M., Zhu K., van de Lagemaat J., Luther J. M., Beard M. C. (2016). Nat. Photonics.

[cit11] Xing G., Mathews N., Sun S., Lim S. S., Lam Y. M., Gratzel M., Mhaisalkar S., Sum T. C. (2013). Science.

[cit12] Sum T. C., Mathews N., Xing G., Lim S. S., Chong W. K., Giovanni D., Dewi H. A. (2016). Acc. Chem. Res..

[cit13] Li M., Bhaumik S., Goh T. W., Kumar M. S., Yantara N., Grätzel M., Mhaisalkar S., Mathews N., Sum T. C. (2017). Nat. Commun..

[cit14] Fang H.-H., Adjokatse S., Shao S., Even J., Loi M. A. (2018). Nat. Commun..

[cit15] Zhou Y., Chen J., Bakr O. M., Sun H.-T. (2018). Chem. Mater..

[cit16] Akkerman Q. A., Meggiolaro D., Dang Z., De Angelis F., Manna L. (2017). ACS Energy Lett..

[cit17] Parobek D., Roman B. J., Dong Y., Jin H., Lee E., Sheldon M., Son D. H. (2016). Nano Lett..

[cit18] Liu W., Lin Q., Li H., Wu K., Robel I., Pietryga J. M., Klimov V. I. (2016). J. Am. Chem. Soc..

[cit19] Guria A. K., Dutta S. K., Das Adhikari S., Pradhan N. (2017). ACS Energy Lett..

[cit20] Ji S., Yuan X., Cao S., Ji W., Zhang H., Wang Y., Li H., Zhao J., Zou B. (2020). J. Phys. Chem. Lett..

[cit21] Meng J., Lan Z., Abdellah M., Yang B., Mossin S., Liang M., Naumova M., Shi Q., Gutierrez Alvarez S. L., Liu Y., Lin W., Castelli I. E., Canton S. E., Pullerits T., Zheng K. (2020). J. Phys. Chem. Lett..

[cit22] König D., Casalenuovo K., Takeda Y., Conibeer G., Guillemoles J. F., Patterson R., Huang L. M., Green M. A. (2010). Phys. E.

[cit23] Protesescu L., Yakunin S., Bodnarchuk M. I., Krieg F., Caputo R., Hendon C. H., Yang R. X., Walsh A., Kovalenko M. V. (2015). Nano Lett..

[cit24] Luo B., Li F., Xu K., Guo Y., Liu Y., Xia Z., Zhang J. Z. (2019). J. Mater. Chem. C.

[cit25] Sikora M., Kapusta C., Kníźek K., Jirák Z., Autret C., Borowiec M., Oates C. J., Procházka V., Rybicki D., Zajac D. (2006). Phys. Rev. B: Condens. Matter Mater. Phys..

[cit26] Thibault-starzyk F., Ristic A., Rajic N. (2003). Scanning.

[cit27] Resasco J., Dasgupta N. P., Rosell J. R., Guo J., Yang P. (2014). J. Am. Chem. Soc..

[cit28] Ressler T., Brock S. L., Wong J., Suib S. L. (1999). J. Synchrotron Radiat..

[cit29] Farges F. (2005). Phys. Rev. B: Condens. Matter Mater. Phys..

[cit30] Chen J. M., Lee J. M., Huang S. W., Lu K. T., Jeng H. T., Chen C. K., Haw S. C., Chou T. L., Chen S. A., Hiraoka N., Ishii H., Tsuei K. D., Yang T. J. (2010). Phys. Rev. B: Condens. Matter Mater. Phys..

[cit31] Hozoi L., de Vries A. H., Broer R. (2001). Phys. Rev. B: Condens. Matter Mater. Phys..

[cit32] Hu Y., Borca C. N., Kleymenov E., Nachtegaal M., Delley B., Janousch M., Dönni A., Tachibana M., Kitazawa H., Takayama-Muromachi E., Kenzelmann M., Niedermayer C., Lippert T., Wokaun A., Schneider C. W. (2012). Appl. Phys. Lett..

[cit33] Croft M., Sills D., Greenblatt M., Lee C. (1997). Phys. Rev. B: Condens. Matter Mater. Phys..

[cit34] De Vries A. H., Hozoi L., Broer R. (2002). Int. J. Quantum Chem..

[cit35] Chaboy J., Prieto C., Hernando M., Parras M., González-Calbet J. (2006). Phys. Rev. B: Condens. Matter Mater. Phys..

[cit36] Price M. B., Butkus J., Jellicoe T. C., Sadhanala A., Briane A., Halpert J. E., Broch K., Hodgkiss J. M., Friend R. H., Deschler F. (2015). Nat. Commun..

[cit37] Yogamalar N. R., Chandra Bose A. (2011). Appl. Phys. A.

[cit38] Cunningham P. D., Hanbicki A. T., McCreary K. M., Jonker B. T. (2017). ACS Nano.

[cit39] Li M., Fu J., Xu Q., Sum T. C. (2019). Adv. Mater..

[cit40] Cao W., Yuan L., Patterson R., Wen X., Tapping P. C., Kee T., Veetil B. P., Zhang P., Zhang Z., Zhang Q., Reece P., Bremner S., Shrestha S., Conibeer G., Huang S. (2017). Nanoscale.

[cit41] Klimov V., Haring Bolivar P., Kurz H. (1995). Phys. Rev. B: Condens. Matter Mater. Phys..

[cit42] Wang L., Chen Z., Liang G., Li Y., Lai R., Ding T., Wu K. (2019). Nat. Commun..

[cit43] Sekiguchi F., Hirori H., Shimazaki A., Nakamura T., Wakamiya A., Kanemitsu Y. (2021). Phys. Rev. Lett..

[cit44] Jia X., Jiang J., Zhang Y., Qiu J., Wang S., Chen Z., Yuan N., Ding J. (2018). Appl. Phys. Lett..

[cit45] Chen J., Messing M. E., Zheng K., Pullerits T. (2019). J. Am. Chem. Soc..

[cit46] Feldmann S., Gangishetty M. K., Bravić I., Neumann T., Peng B., Winkler T., Friend R. H., Monserrat B., Congreve D. N., Deschler F. (2021). J. Am. Chem. Soc..

[cit47] Kawai H., Giorgi G., Marini A., Yamashita K. (2015). Nano Lett..

[cit48] Even J., Pedesseau L., Katan C. (2014). J. Phys. Chem. C.

[cit49] Rudin B. S. S., Reinecke T. L. (1990). Phys. Rev. B: Condens. Matter Mater. Phys..

[cit50] Wright A. D., Verdi C., Milot R. L., Eperon G. E., Pérez-Osorio M. A., Snaith H. J., Giustino F., Johnston M. B., Herz L. M. (2016). Nat. Commun..

[cit51] Kahmann S., Loi M. A. (2019). J. Mater. Chem. C.

[cit52] Conibeer G. J., König D., Green M. A., Guillemoles J. F. (2008). Thin Solid Films.

[cit53] Shafique A., Shin Y. H. (2017). Phys. Chem. Chem. Phys..

[cit54] Straus D. B., Hurtado Parra S., Iotov N., Gebhardt J., Rappe A. M., Subotnik J. E., Kikkawa J. M., Kagan C. R. (2016). J. Am. Chem. Soc..

