# Supplementary material for: Optimizing the quasi-equilibrium state of hot carriers in all-inorganic lead halide perovskite nanocrystals through Mn doping: fundamental dynamics and device perspectives
Source: Chem Sci. 2022 Jan 14;13(6):1734–45. doi: 10.1039/d1sc05799e (PMC8827087; doi:10.1039/d1sc05799e)
Supplement: SC-013-D1SC05799E-s001 [file SC-013-D1SC05799E-s001.pdf]

Electronic Supplementary Material (ESI) for Chemical Science.
This journal is © The Royal Society of Chemistry 2022

Supporting Information

Optimizing the quasi-equilibrium state of hot carriers in all-inorganic lead halide perovskite nanocrystals through Mn-doping: fundamental dynamics and device perspectives

Jie Meng[†,1], Zhenyun Lan[‡,1], Weihua Lin[#], Mingli Liang[†], Xianshao Zou[#], Qian Zhao[†], Huifang Geng[¶], Ivano E. Castelli[‡], Sophie E. Canton[§], Tönu Pullerits[#], and Kaibo Zheng[*,†,#]

[†]Department of Chemistry, Technical University of Denmark, DK-2800 Kongens Lyngby, Denmark;

[‡]Department of Energy Conversion and Storage, Technical University of Denmark, DK-2800 Kongens Lyngby, Denmark;

[#]Chemical Physics and NanoLund, Lund University, Box 124, 22100 Lund, Sweden;

[¶]Ultrafast Electron Microscopy Laboratory, The MOE Key Laboratory of Weak-Light Nonlinear Photonics, School of Physics, Nankai University, Tianjin 300071, China;

[§] European XFEL, Holzkoppel 4, 22869 Schenefeld, Germany.

[*]Corresponding Author

Kaibo Zheng: kzheng@kemi.dtu.dk, kaibo.zheng@chemphys.lu.se.

[1] This authors contribute equally in this work

Experimental Section

Materials and Chemicals. Cesium carbonate (Cs_2CO_3, ReagentPlus 99%, Sigma-Aldrich), Lead iodide (PbI_2, 99%, Sigma-Aldrich), Manganese iodide (MnI_2 anhydrous, 99.99% trace metals basis, Sigma-Aldrich), Oleylamine (OAm, technical grade 70%, Sigma-Aldrich), Oleic acid (OA, technical grade 90%, Sigma-Aldrich), 1-Octadecene (ODE, technical grade 90%, Sigma-Aldrich), Toluene (anhydrous 99.8%, Sigma-Aldrich).

Preparation of Cs-OA. Cs_2CO_3 (0.407 g), OA (1.5 mL), and ODE (20 mL) were added to a 50-mL 3-neck round-bottomed flask, evacuated and refilled with argon, dried at 120 °C for 30 min, then heated to 150 °C for at least 10 minutes until all Cs_2CO_3 reacted with OA before using.

Synthesis of Mn-doped $CsPbI_3$ NCs. Typically, PbI_2 (0.087 g, 0.188 mmol), MnI_2 (precusor: 0-0.01-0.02-0.03 g), OAm (0.5 mL), OA (0.5 mL), ODE (5 mL) were added to a 25-mL 3 neck round bottom flask and were evacuated and refilled with Ar followed by heating the solution to 120 °C for 30 minutes. Then dried OAm (0.5 mL) and dried OA (0.5 mL) were subsequently injected to solubilize the solution. The solution was then increased to 160 °C. At 160 °C, the Cs-oleate (0.4 mL) was swiftly injected and after 1 minute the solution was cooled with an ice/water bath. After reaction, the aggregated NCs were centrifuged at 6000 rpm for 5 min. After centrifugation, the supernatant was discarded and the precipitate was redispersed in dried toluene. And then, the solution will be centrifuged at 6000 rpm for 5 min and the supernatant will be redispersed in toluene for further measurement. The dopant concentration (0%, 5%, 7%, 10%) are obtained by Inductively coupled plasma mass spectrometry (ICP-MS).

Characterization. The absorption spectra were measured in a UV-Vis absorption spectrophotometer from Agilent Technologies (Santa Clara, USA). Steady-state

photoluminescence was measured using a FluoroMax@-4 spectrofluorometer (HORIBA JOBIN YVON, Inc., Edison, NJ) with the excitation at 500 nm. Transmission electron microscopy (TEM) imaging was conducted on Tecnai G^2 T20 TEM. The Mn doping concentration was determined by coupled mass spectrometry (*ICP*-MS)

TA measurement. The transient absorption (TA) experiments were performed on a femtosecond pump-probe setup. Laser pulses (800 nm, 80 fs pulse length, 0.5 kHz repetition rate) were generated by a femtosecond oscillator (Mai Tai SP, both Spectra Physics). The pump pulses at 400 nm, 500 nm and 570 nm were generated by an optical parametric amplifier (Topas, Light Conversion). For the probe, we used the super-continuum generation from a thin CaF_2 plate. The mutual polarization between pump and probe beams was set to the magic angle (54.7°) by placing a Berek compensator in the pump beam. The probe pulse and the reference pulse were dispersed in a spectrograph and detected by a diode array (Pascher Instruments). Excitation power and spot size measurements were used to determine the excitation fluence. To prevent sample degeneration, several strategies have been carried out: 1) the sample will be prepared only before spectroscopic measurement. 2) For each measurement, we will change the fresh sample. After measurement, we also checked the UV-vis absorption spectroscopies to check the samples.

Absorption cross-section determination. NCs can be excited to multiple exciton states by high excitation fluence. With a usual assumption, the initially generated multiple exciton population follows the Poissonian distribution

$$P_N = \frac{e^{-\langle N \rangle} \cdot \langle N \rangle^N}{N!} \qquad \text{(S1)}$$

where $\langle N \rangle$ is the average number of excitons per NCs, N is the number of excitons, and P_N is the fraction of NCs with N excitons. We can use $\langle N \rangle = \sigma \cdot I$ to present the average number of excitons

per NCs, where σ is the absorption cross-section at the excitation wavelength and I is the excitation intensity in units of the number of photons per pulse per excitation area. From equation S1, we can calculate the fraction of excited NCs, P_{exc}, as:

$$P_{exc}=\sum_{N=1}^{\infty} P_N=1-P_0=1-e^{-\langle N\rangle}=1=1-e^{-\sigma\cdot I} \qquad \text{(S2)}$$

If we know P_{exc}, σ can be calculated from (S2). We obtain P_{exc} by measuring the excitation intensity dependence of the late-time region signal ($t > 1\ ns$), which corresponds to the last remaining exciton after the Auger process. Due to multiple excitations generated at high pump intensity excitation in NCs is rapidly lost via Auger process leaving only one excitation at latetime region ($t > 1\ ns$). The signal ΔA_0 (I, $t > 1\ ns$) intensity can be rescaled to the corresponding signal at $t=0$, which we call $\Delta A_0(I)$. We use the lowest excitation intensity as reference excitation intensity and corresponding average number of excitons per NCs, $\langle N\rangle_0$.as reference number of excitons per NCs.

$$\Delta A_0(I) = \frac{\Delta A(I,t>1ns)}{e^{-t/\tau}} = \Delta A_{0,max}\cdot\left(1-e^{-(I/I_0)\cdot\langle N\rangle_0}\right) \qquad \text{(S3)}$$

$\Delta A_{0,}$ denotes the largest possible single-exciton signal rescaled to t=0.

Based on the rescaled signal $\Delta A_0(I)$, we perform exponential fit to equation S3. From the fitting, we get the value of $\langle N\rangle_0$, and absorption cross-section σ of Mn doped $CsPbI_3$ NCs at 400 nm (3.1 eV) were calculated. The absorption cross-section σ at 500 nm and 570 nm were calculated based on the values of σ at 400 nm and absorption spectra.

Power loss of the hot carriers. The power loss of hot carriers is obtained from the time-dependent hot carriers temperature Tc using follow equation. k_B is Boltzmann constant.

$$P = \frac{d(\frac{3}{2}k_B T_c)}{dt} \quad \text{(S4)}$$

Average lifetime calculation

A multi-exponential model was used to fit the cooling process curves. The model is described as follows:

$$f(x) = A + \Sigma B_i \exp(-x/t_i)$$

where t_i represents the time constants and B_i represents the amplitudes of the fast and slow components, respectively. The average lifetime was calculated as follows:

$$t_{ave} = (\Sigma B_i t_i^2)/(\Sigma B_i t_i)$$

DFT calculation

The density functional theory (DFT) calculations were performed using Perdew-Burke-Ernzerhof (PBE) exchange correlation functional,[1] as implemented in the Vienna ab initio package (VASP).[2] The electronic wave functions were calculated by using projected augmented wave (PAW)[3] with a plane-wave cutoff of 520 eV. Due to the heavy Pb atom in $CsPbCl_3$, spin-orbit coupling (SOC) was included for the electronic properties calculation. The unit cell $CsPbCl_3$ structure is cubic containing 5 atoms with lattice parameter of 6.401 Å. A single Pb atom was replaced by a Mn atom in the 2 x 2 x 2 supercell corresponding to Mn concentration of 6.25%. Phonon spectrum was calculated using the DFPT method by the Phonon code. The Brillouin zone samplings were carried out with a Γ-center 4 x 4 x 4 k-point mesh. The atomic forces were relaxed to be less than

0.01 Å^{-1}. While for the HSE06 band structure calculation, the plane wave cutoff was set as 300 eV and the Brillouin zone was sampled by 2 x 2 x 2 Γ-centered k-point grid. It should also be noted that the imaginary phonon modes with a negative frequency result from the metastable lattice of the perovskite where the permanent displacement of the atoms is possible through distortions of the crystal lattice.

X-ray absorption measurements

X-ray absorption spectra were acquired at P65 beamline of Petra III, Deutsches Elektronen - Synchrotron (DESY), Hamburg.[4] The beam was monochromatized using Si(111) crystal pair. Higher harmonics rejection was done with two Si-coated mirrors. Solutions of Mn-doped nanoparticles in toluene were filled into 2 mm quartz capillaries (wall thickness 0.02 mm) which were positioned horizontally at 45 degrees to the incoming beam. Beam size on the sample was 2 mm (hor.) * 0.5 mm (ver.). Data were acquired in fluorescence mode using a 4 pixel Silicon drift detector. Spectra were taken in continuous scanning mode. First, damage tests were done by performing 30 s scans in -50 to 100 eV energy range around Mn K-edge. Then, based on the data quality, extended XANES range was chosen for the study: -100 to 300 eV measured for 120 s; many repetitions were done to improve the data quality. Mn foil was installed downstream of the sample for energy calibration. The maximum of first derivative of the foil was calibrated to 6539 eV.

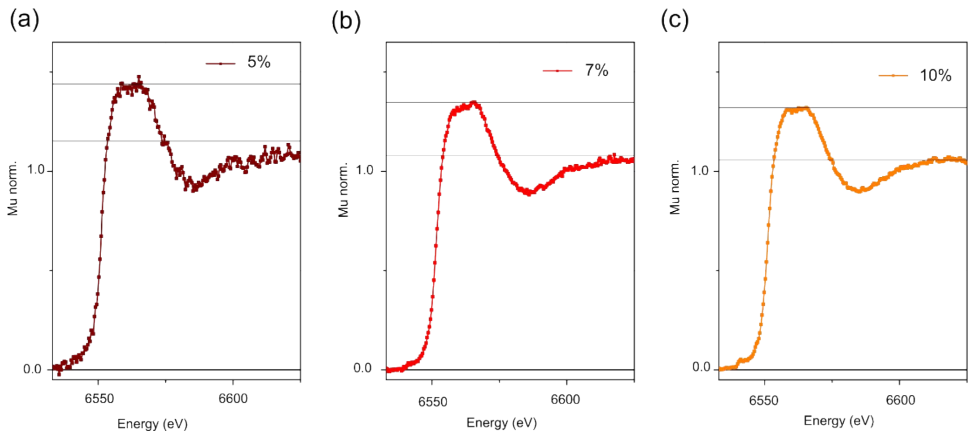

Figure S0. Normalized XANES feature for 5% (a), 7% (b) and 10% (c) doping. The solid lines indicate the maximum intensities and their values at 80% of the maximum in order to estimate the width of the white line features.

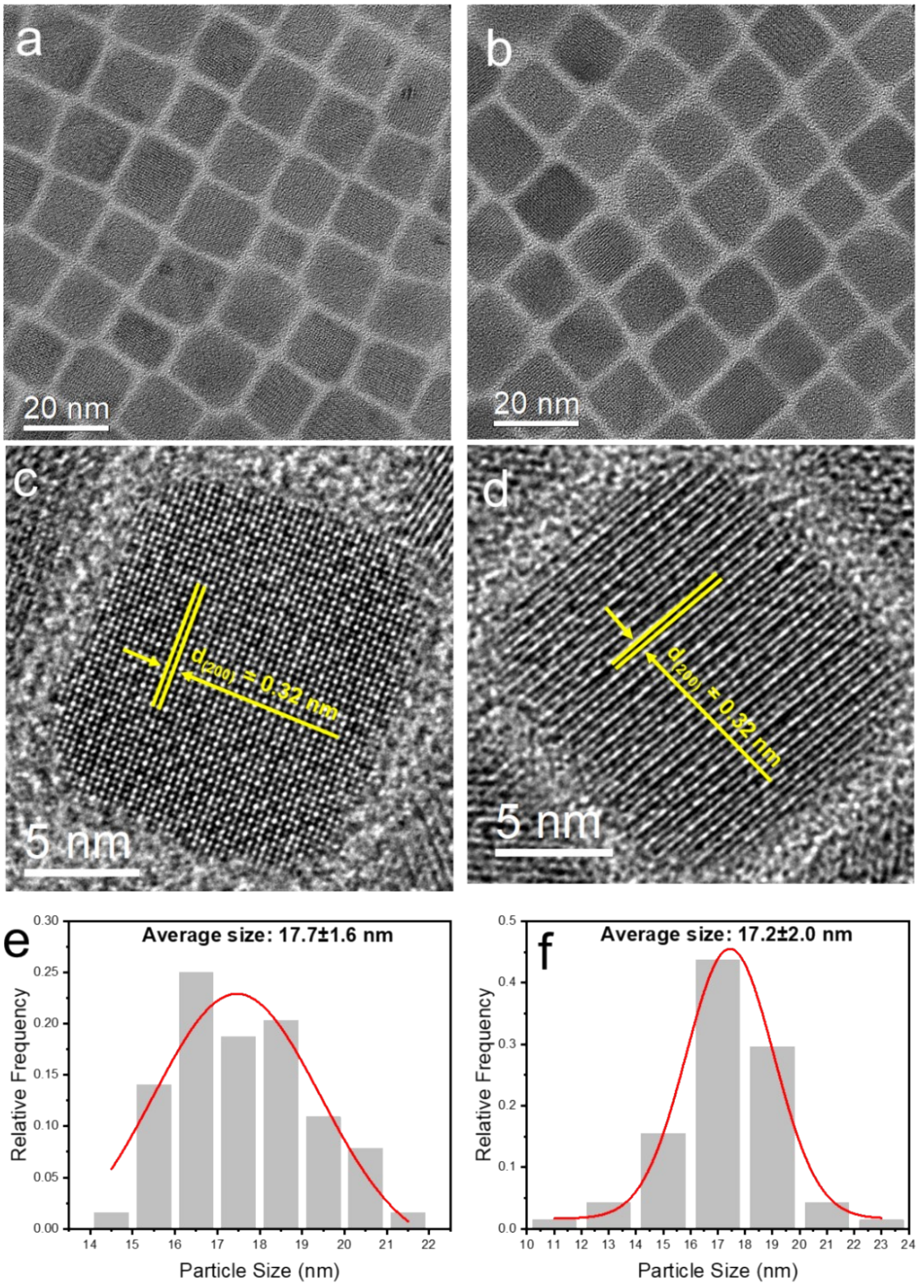

Figure S1. The TEM images and size distribution of samples. The TEM images of (a) undoped $CsPbI_3$ and (b) Mn doped $CsPbI_3$. The HRTEM images of (c) undoped and (d) doped NCs. The corresponding size distribution of (e) undoped CsPbI3 and (f) Mn doped $CsPbI_3$.

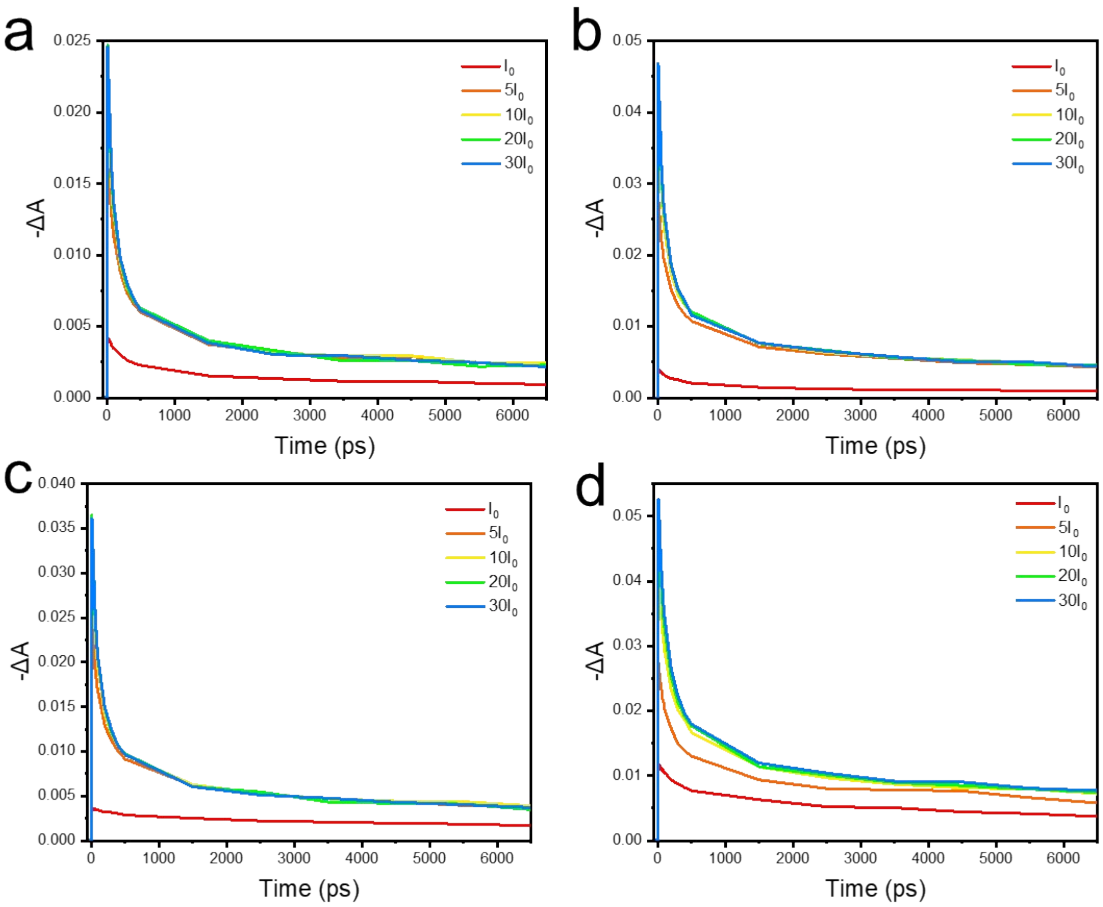

Figure S2. Pump fluence dependent TA kinetics for Mn doped $CsPbI_3$ NCs pumped at 400 nm (3.1 eV) probed at band-edge, I_0=1.603 x 10^{13} photons/cm^2/pulse. (a) 0%, (b) 5%, (c) 7% and (d) 10%.

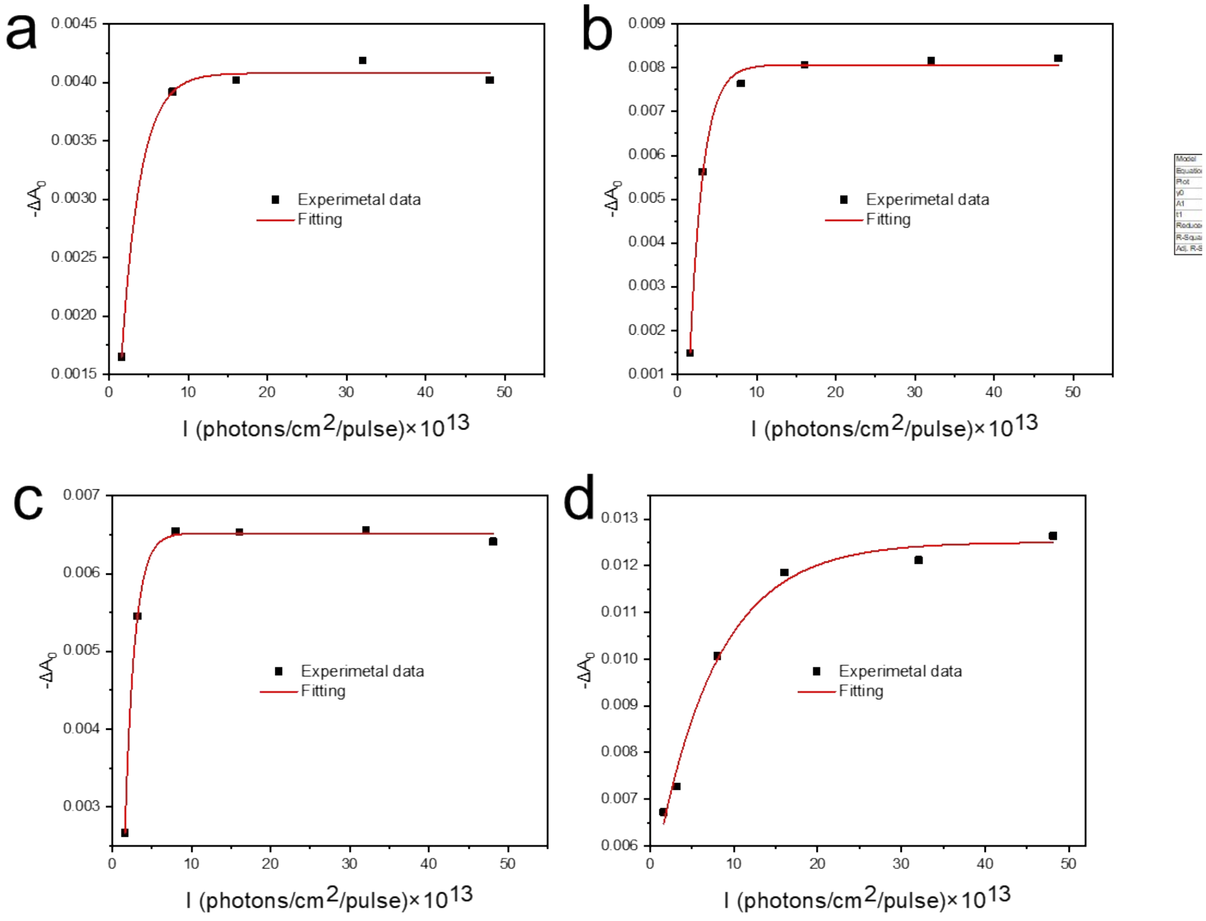

Figure S3. TA signal ΔA_0 rescaled from $\Delta t \geq 1$ ns as a function of pump fluence for Mn doped $CsPbI_3$ NCs pumped at 400 nm (3.1 eV) and probed at band-edge. (a) 0%, (b) 5%, (c) 7% and (d) 10%.

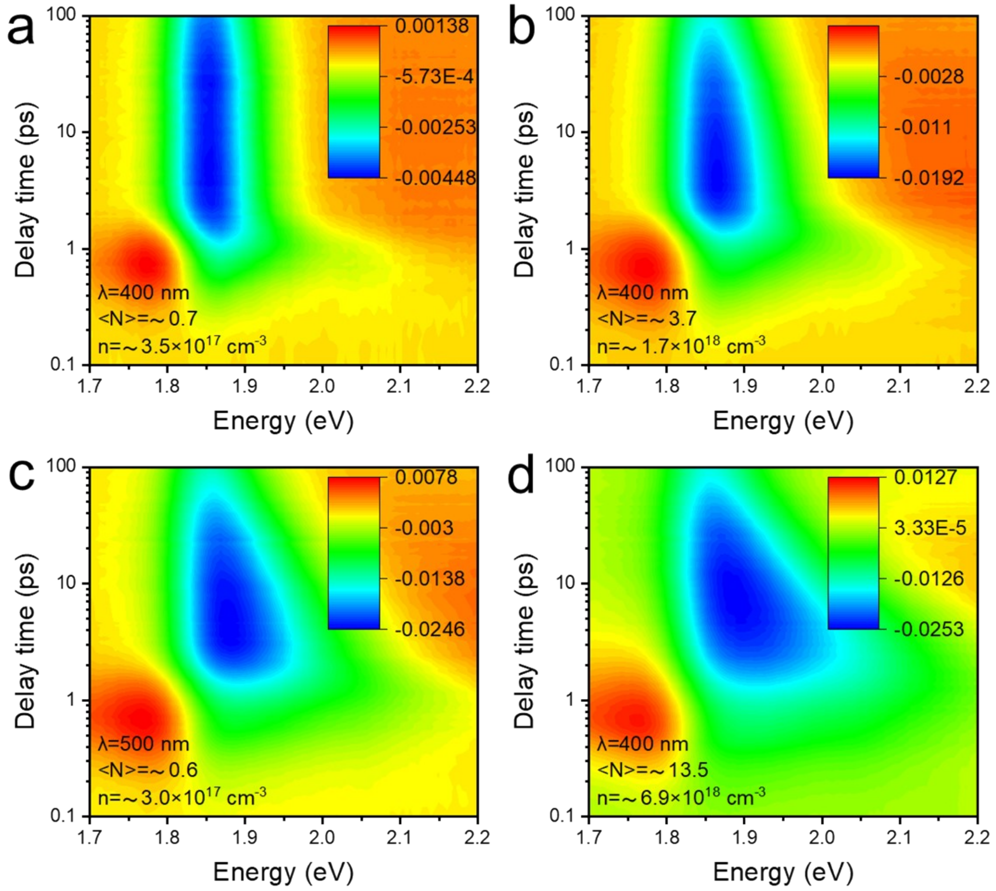

Figure S4. Pseudocolor representation TA spectra of undoped $CsPbI_3$ NCs for 400 nm excitation with different excitation intensities. (a) I1, (b) I2, (c) I3 and (d) I4.

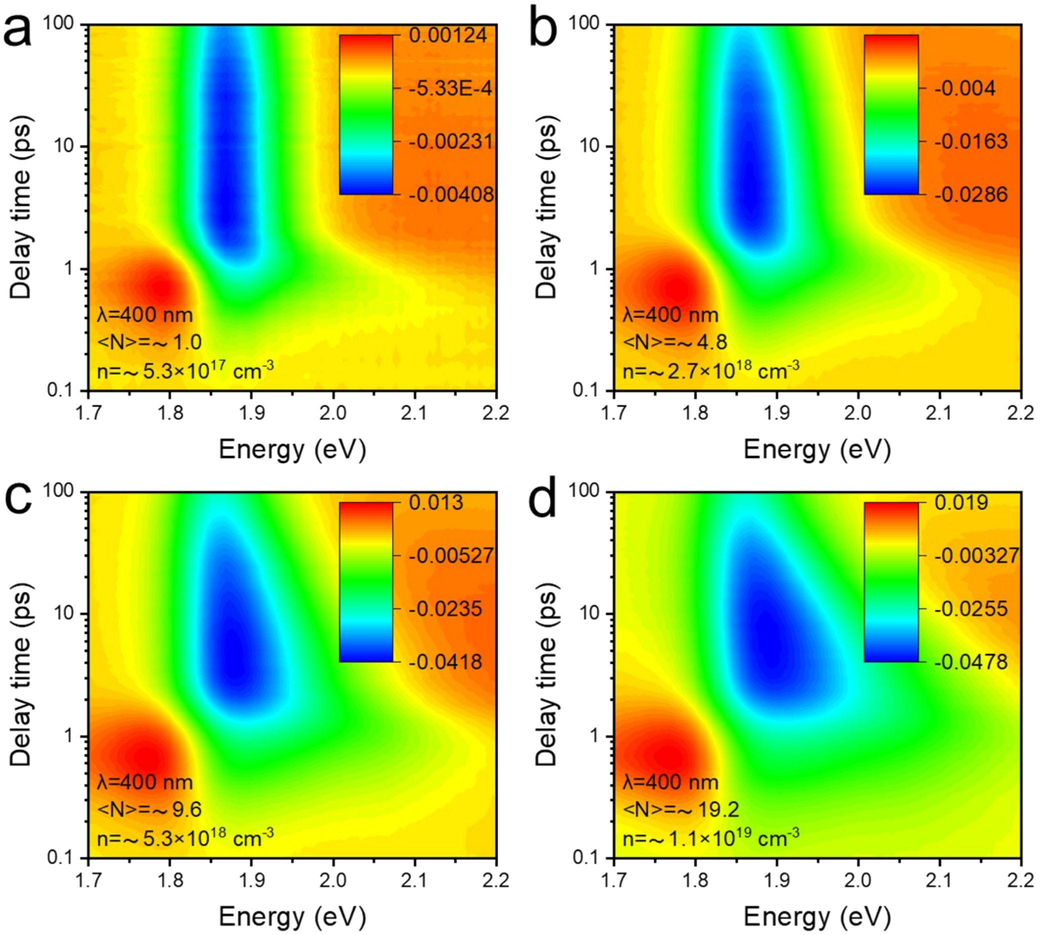

Figure S5. Pseudocolor representation TA spectra of Mn doped $CsPbI_3$ NCs for 400 nm excitation with different excitation intensities. (a) I1, (b) I2, (c) I3 and (d) I4.

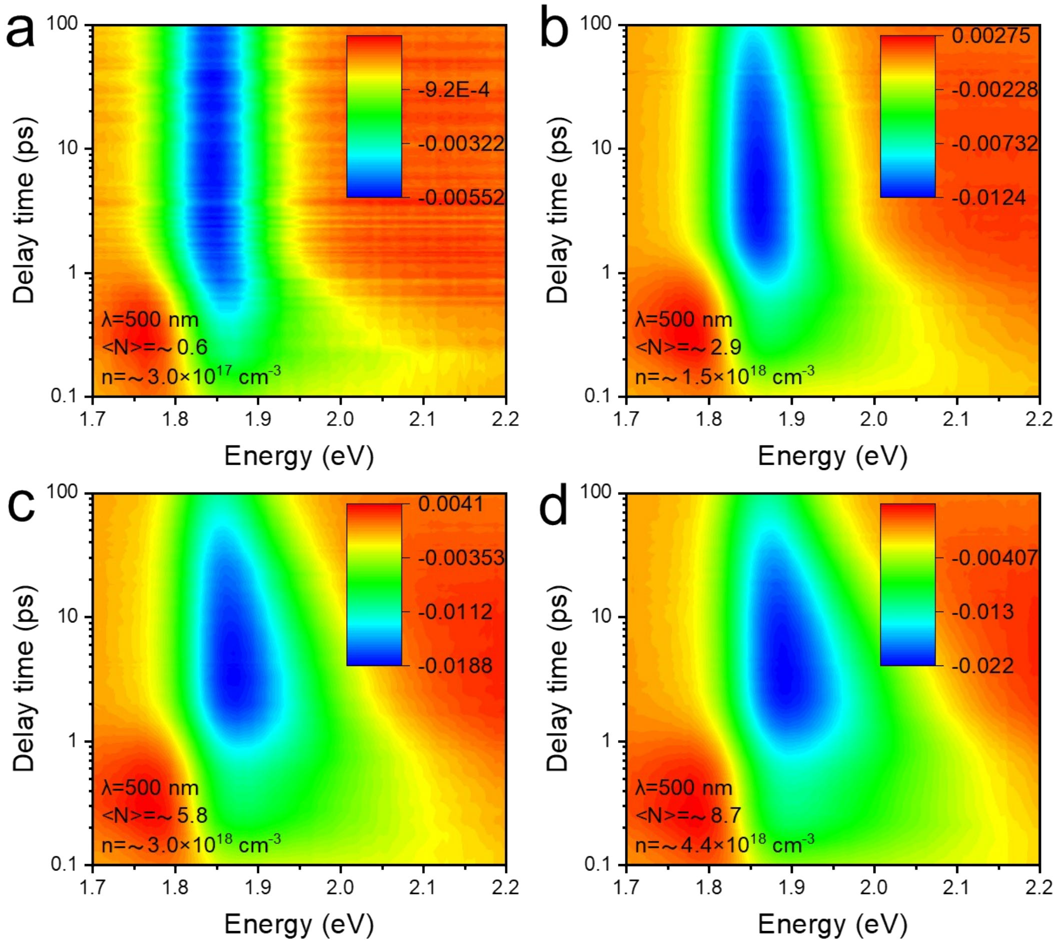

Figure S6. Pseudocolor representation TA spectra of undoped $CsPbI_3$ NCs for 500 nm excitation with different excitation intensities. (a) I1, (b) I2, (c) I3 and (d) I4.

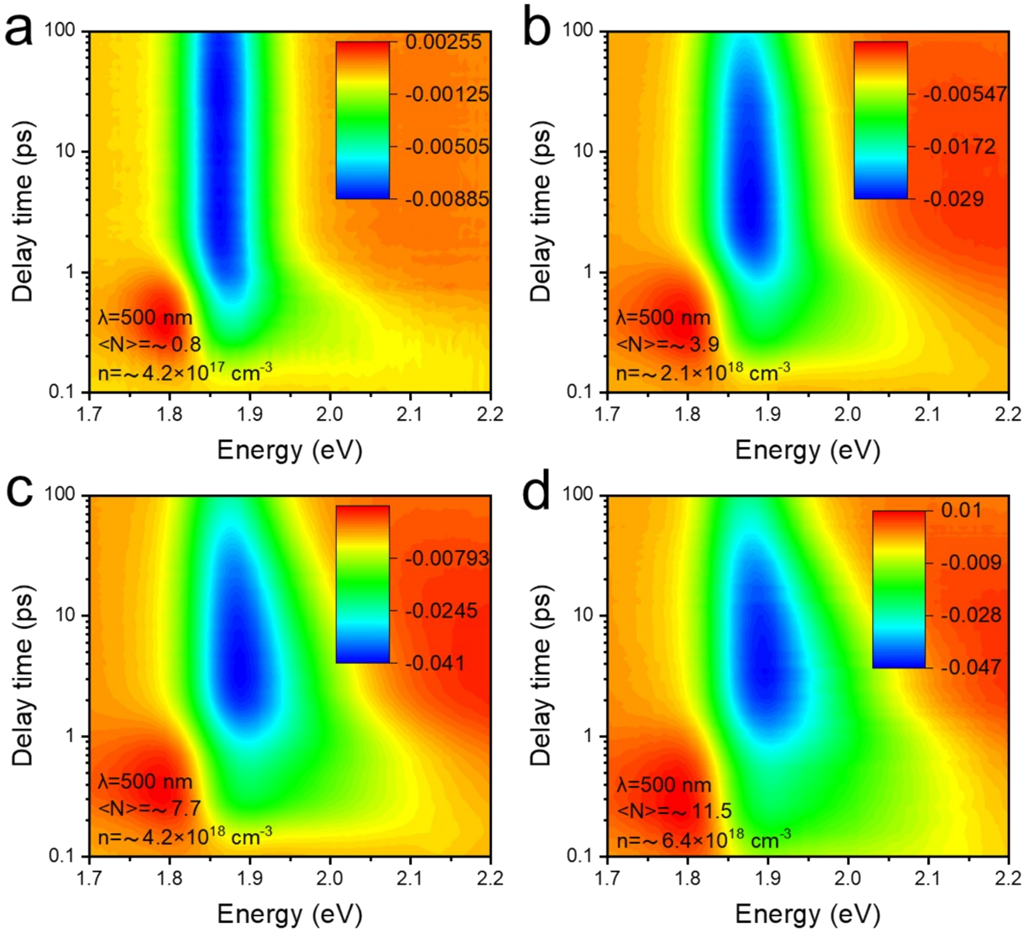

Figure S7. Pseudocolor representation TA spectra of Mn doped $CsPbI_3$ NCs for 500 nm excitation with different excitation intensities. (a) I1, (b) I2, (c) I3 and (d) I4.

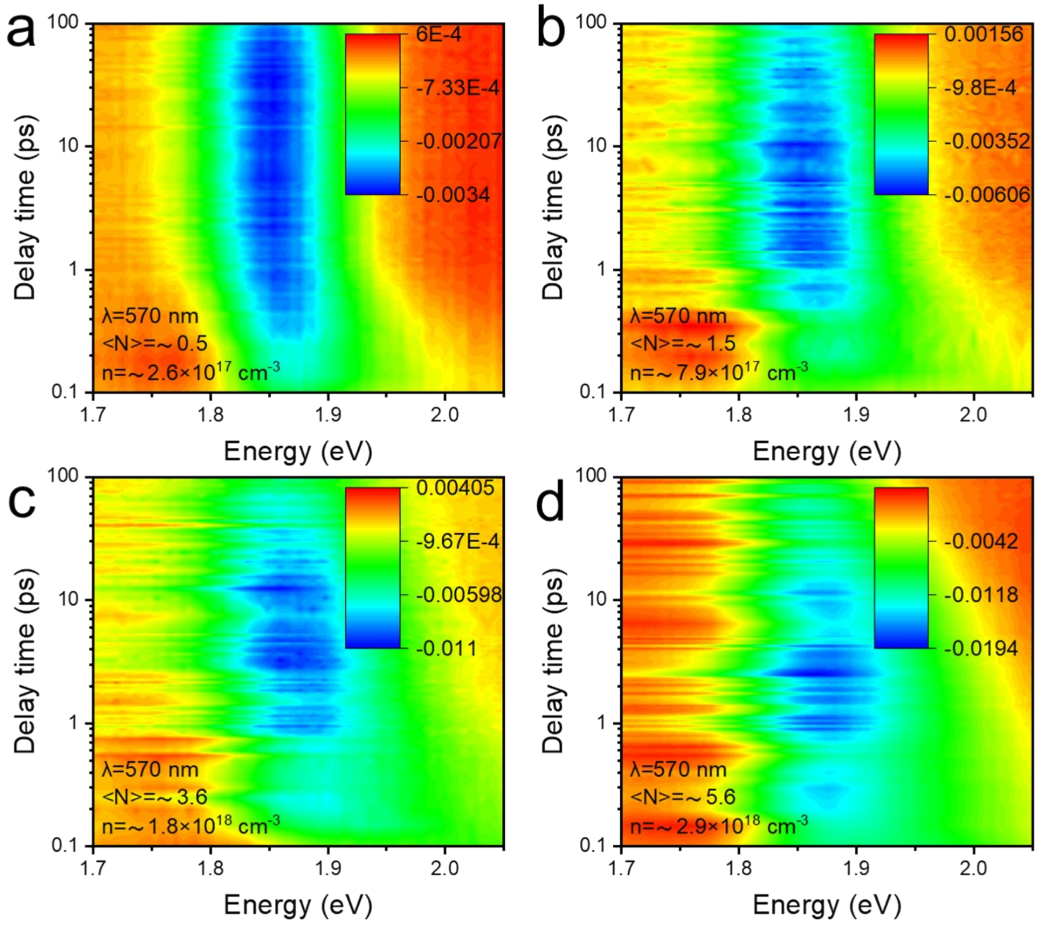

Figure S8. Pseudocolor representation TA spectra of undoped $CsPbI_3$ NCs for 570 nm excitation with different excitation intensities. (a) I1, (b) I2, (c) I3 and (d) I4.

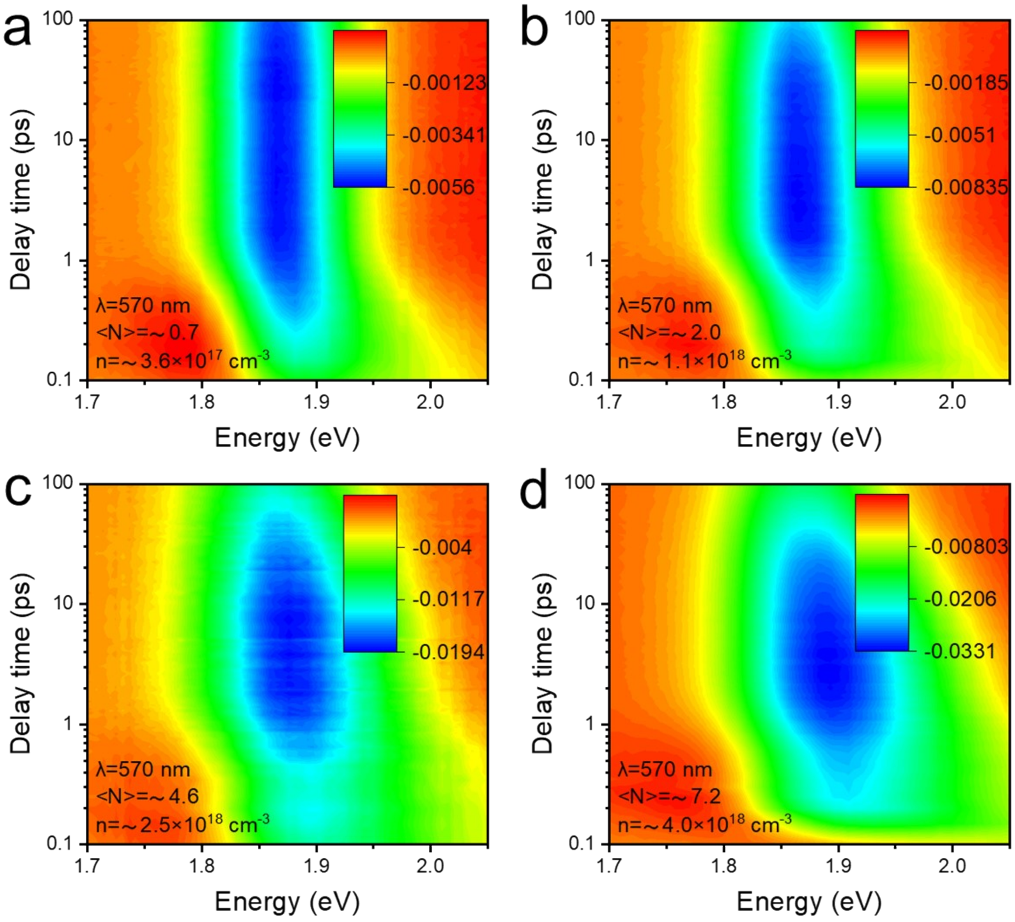

Figure S9. Pseudocolor representation TA spectra of Mn doped $CsPbI_3$ NCs for 570 nm excitation with different excitation intensities. (a) I1, (b) I2, (c) I3 and (d) I4.

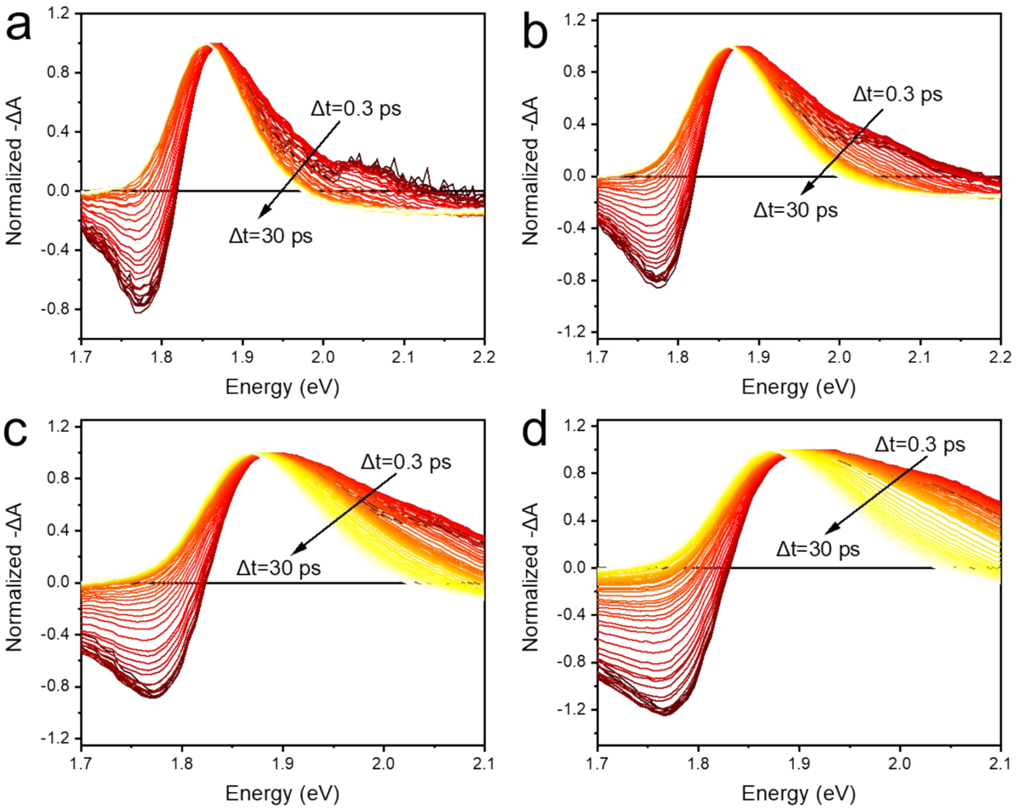

Figure S10. Normalized TA spectra of undoped $CsPbI_3$ NCs under 400 nm excitation with different excitation energies. (a) I1, (b) I2, (c) I3 and (d) I4. The shortest time delay (red) is 0.3 ps and the longest (yellow) is 30 ps.

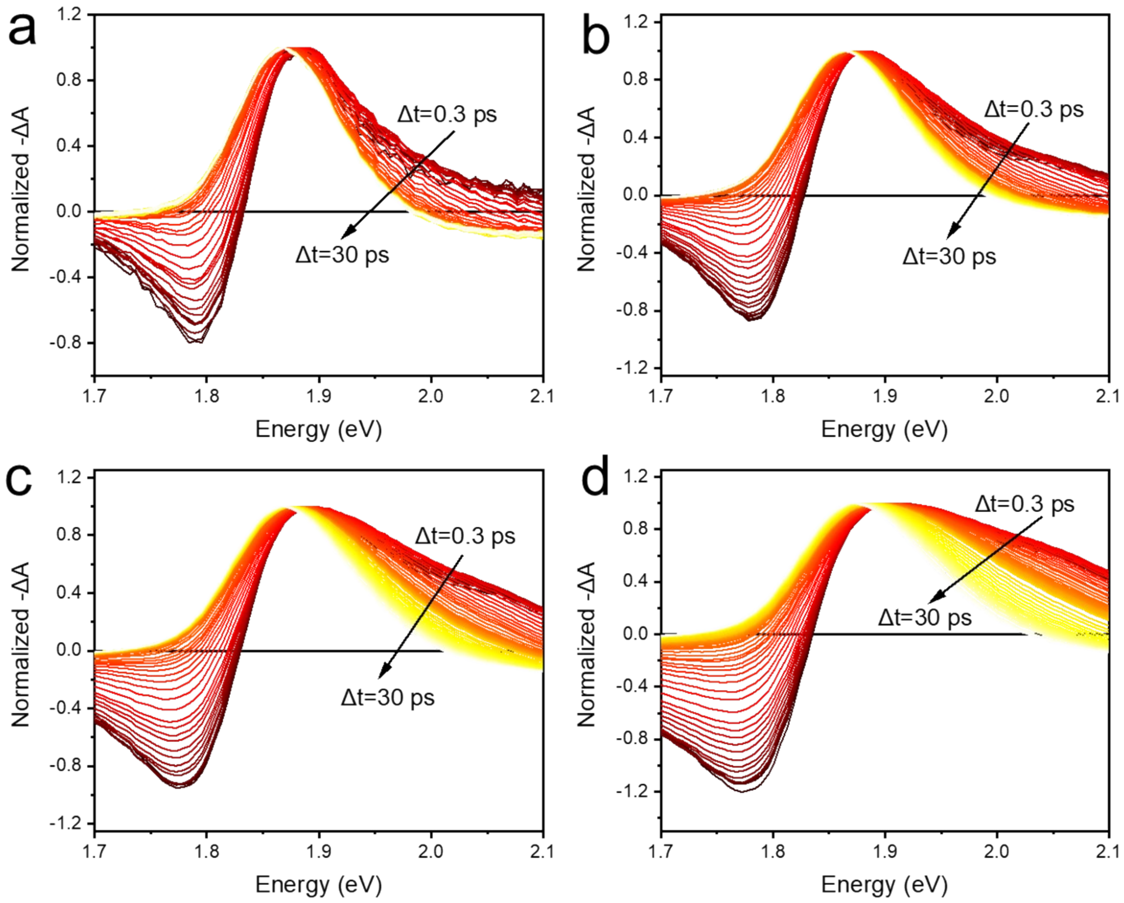

Figure S11. Normalized TA spectra of Mn doped $CsPbI_3$ NCs under 400 nm excitation with different excitation energies. (a) I1, (b) I2, (c) I3 and (d) I4. The shortest time delay (red) is 0.3 ps and the longest (yellow) is 30 ps.

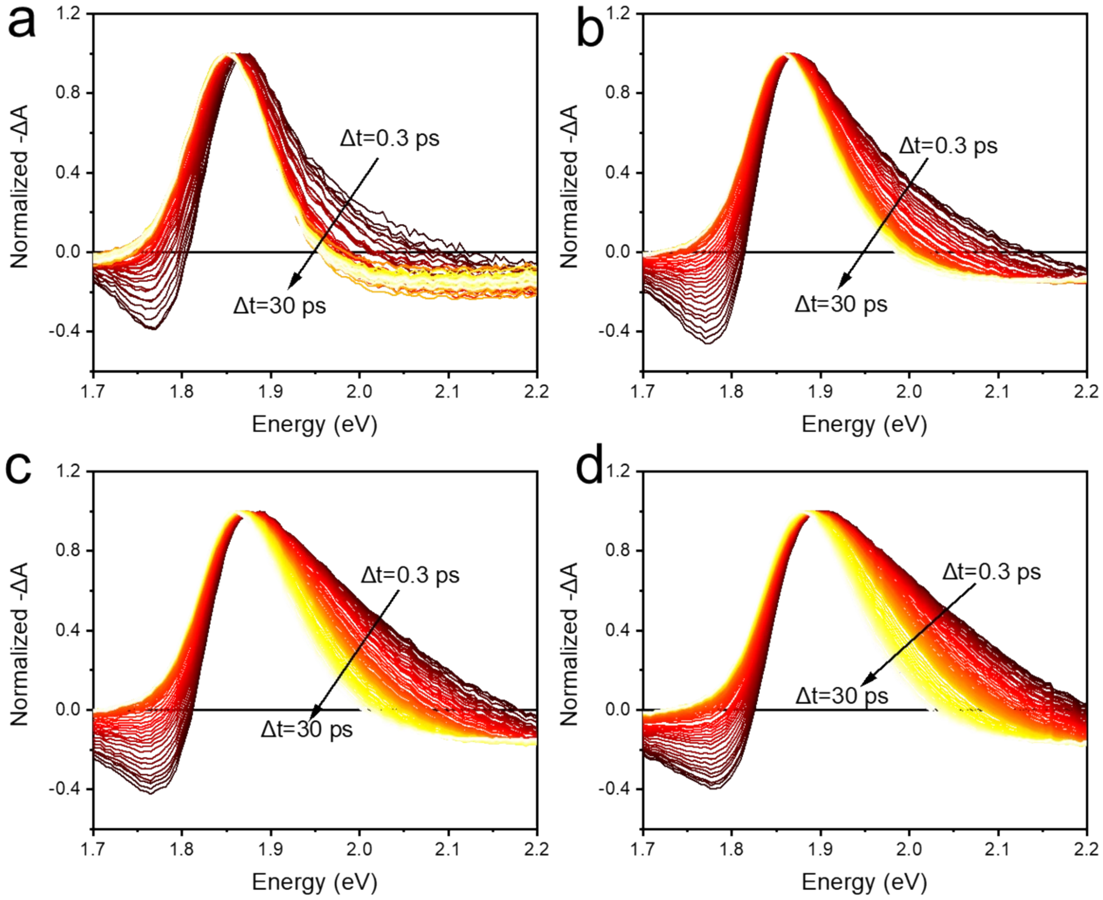

Figure S12. Normalized TA spectra of undoped $CsPbI_3$ NCs under 500 nm excitation with different excitation energies. (a) I1, (b) I2, (c) I3 and (d) I4. The shortest time delay (red) is 0.3 ps and the longest (yellow) is 30 ps.

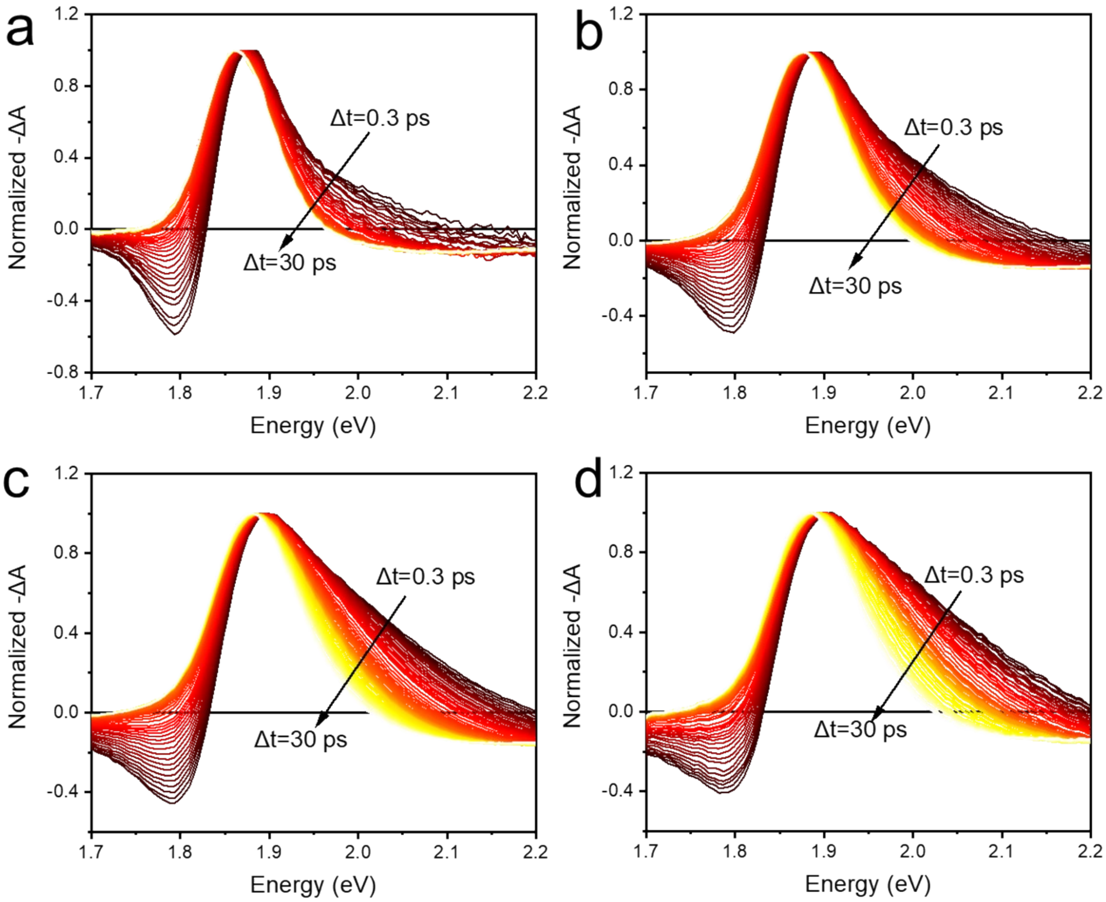

Figure S13. Normalized TA spectra of Mn doped $CsPbI_3$ NCs under 500 nm excitation with different excitation energies. (a) I1, (b) I2, (c) I3 and (d) I4. The shortest time delay (red) is 0.3 ps and the longest (yellow) is 30 ps.

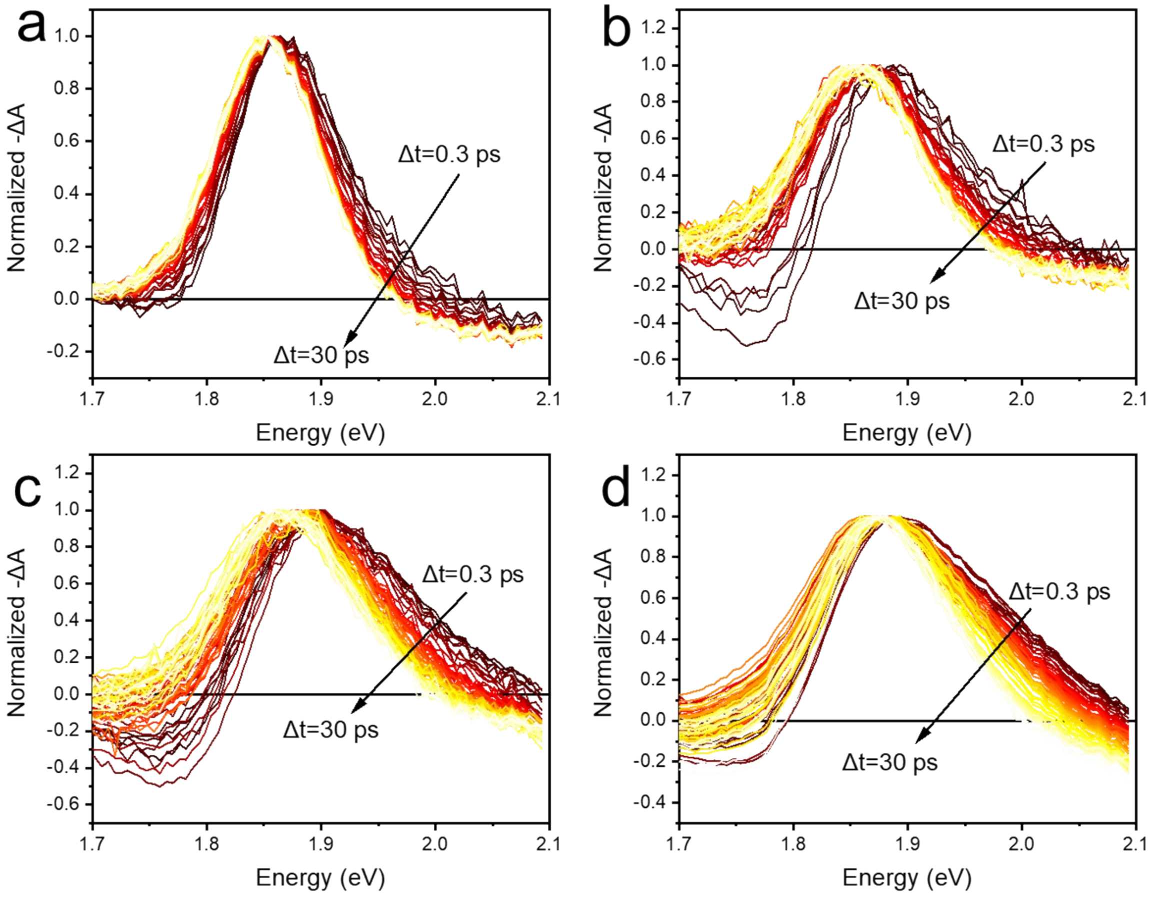

Figure S14. Normalized TA spectra of undoped $CsPbI_3$ NCs under 570 nm excitation with different excitation energies. (a) I1, (b) I2, (c) I3 and (d) I4. The shortest time delay (red) is 0.3 ps and the longest (yellow) is 30 ps.

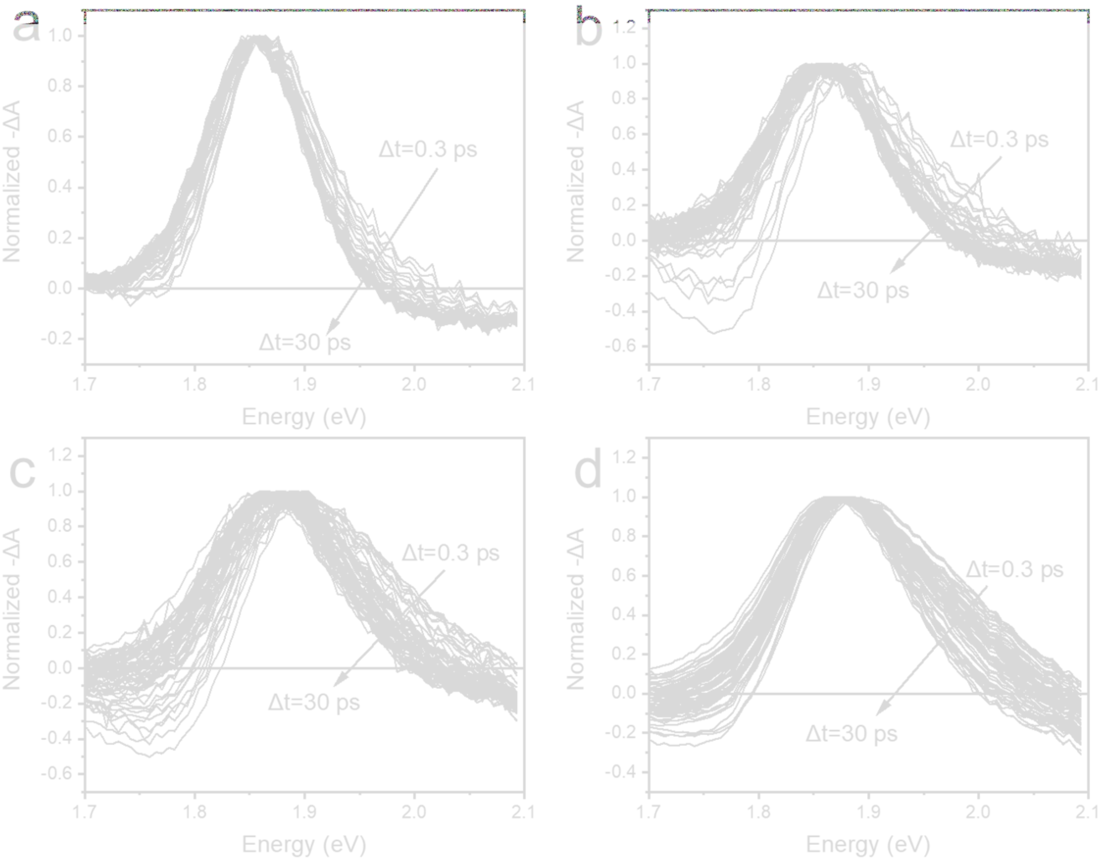

Figure S15. Normalized TA spectra of Mn doped $CsPbI_3$ NCs under 570 nm excitation with different excitation energies. (a) I1, (b) I2, (c) I3 and (d) I4. The shortest time delay (red) is 0.3 ps and the longest (yellow) is 30 ps.

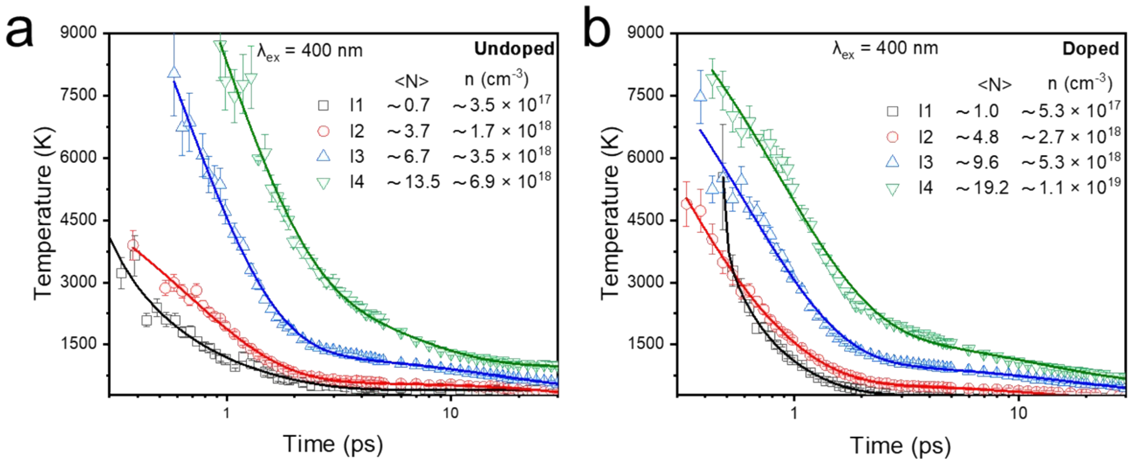

Figure S16. Time-dependent carrier temperature with 400 nm excitation at four different excitation intensities for (a) undoped and (b) Mn doped $CsPbI_3$ NCs.

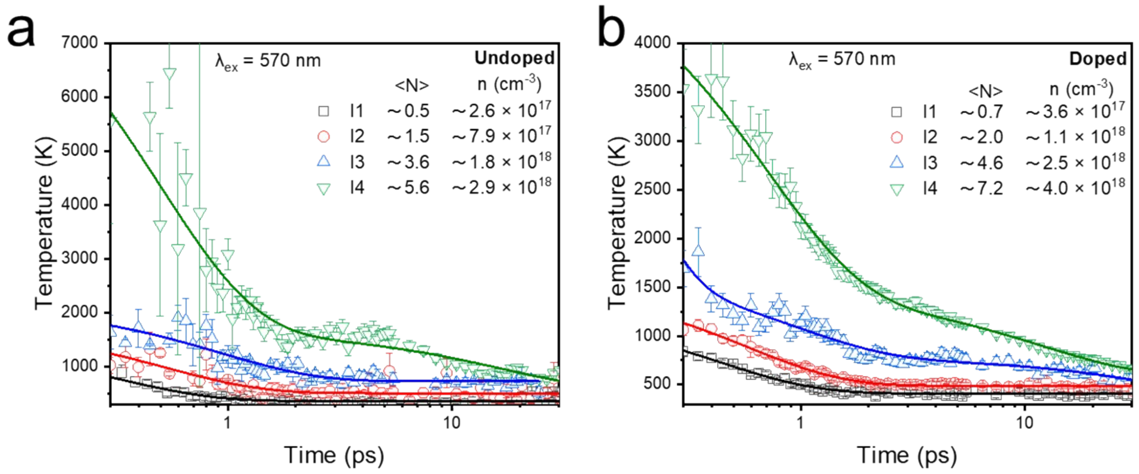

Figure S17. Time-dependent carrier temperature with 570 nm excitation at four different excitation intensities for (a) undoped and (b) Mn doped $CsPbI_3$ NCs.

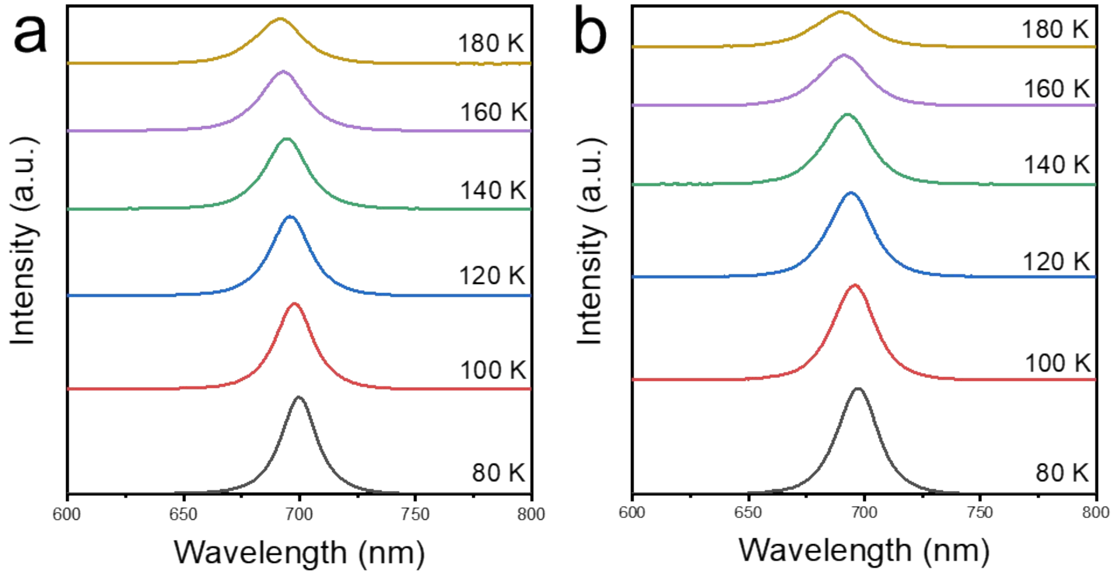

Figure S18. Temperature dependent PL emission spectra of Mn doped $CsPbI_3$ NCs as function of temperature (80 K to 180 K) for different doping concentration. (a) 0% and (b) 5%.

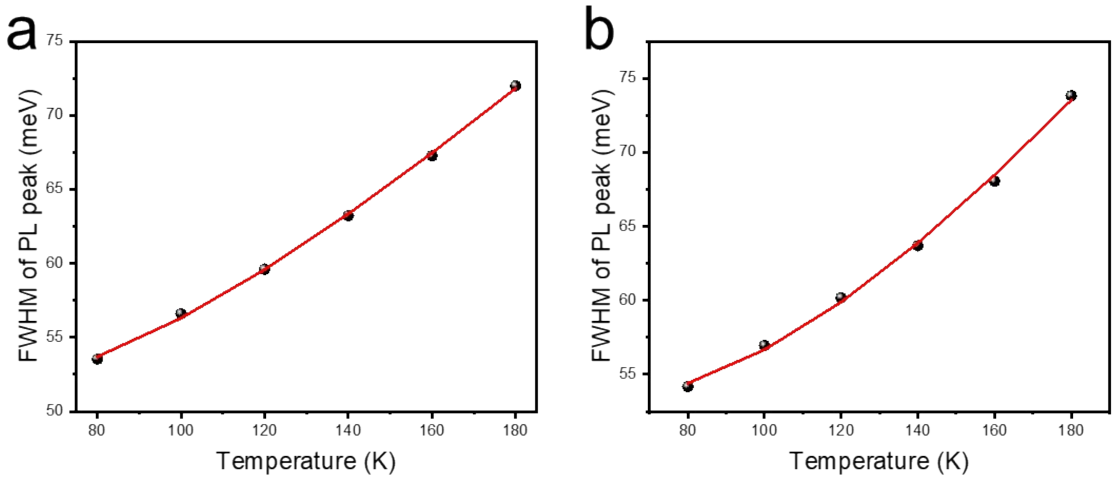

Figure S19. FWHM of the steady-state PL spectra as a function of temperature for of Mn doped $CsPbI_3$ NCs (80 K to 180 K) for different doping concentration. (a) 0% and (b) 5%.

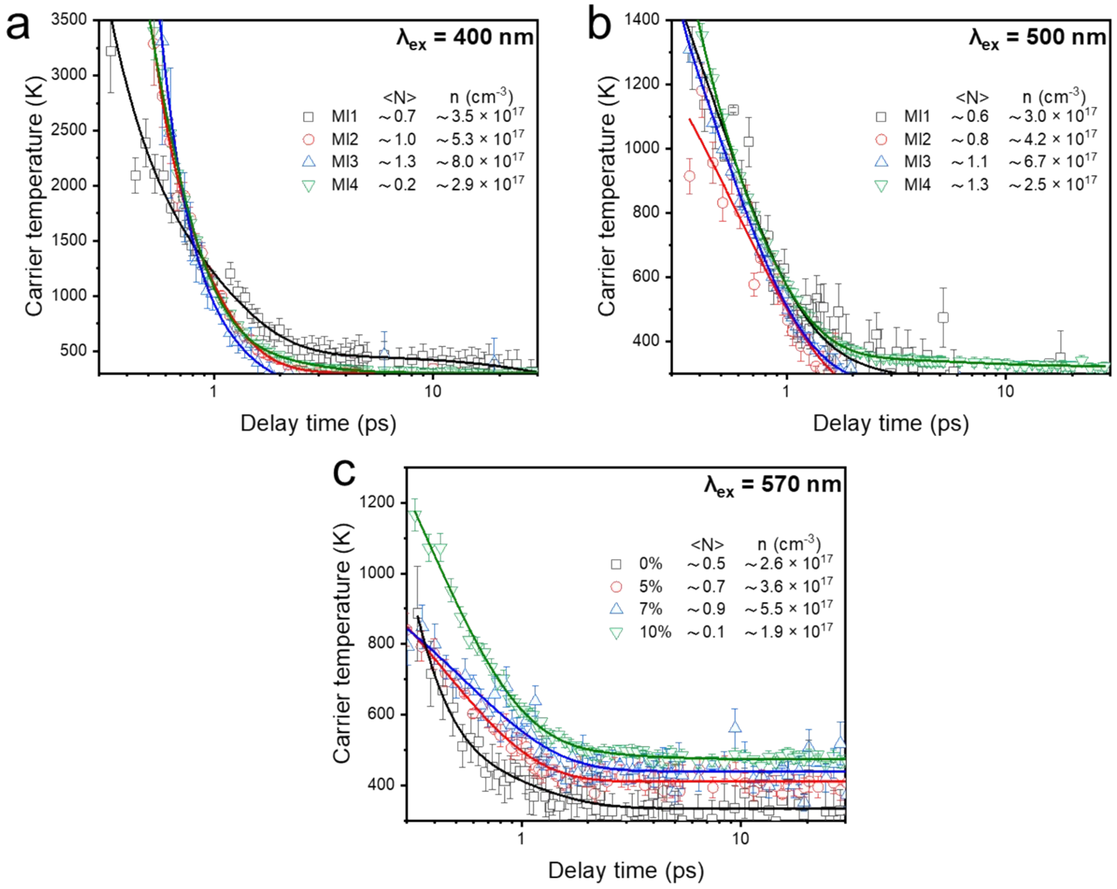

Figure S20. Time-dependent carrier temperature for different doping concentration at low excitation intensity with (a) 400 nm, (b) 500 nm and (c) 570 nm excitation energy.

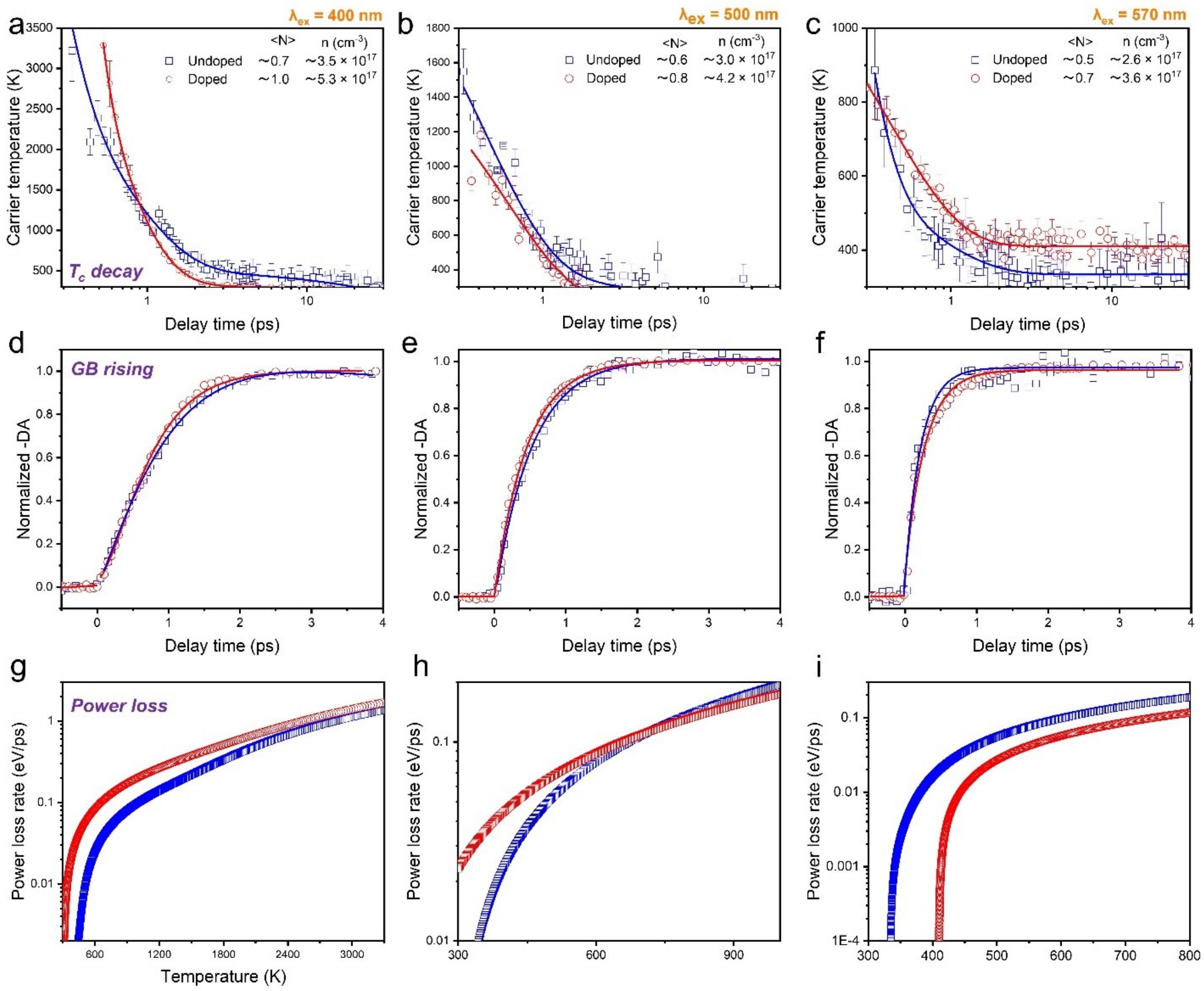

Figure S21. Time-dependent carrier temperature for undoped and Mn-doped $CsPbI_3$ NCs at low excitation density (<N> < 1) with (a) 400 nm, (b) 500 nm and (c) 570 nm excitation energy. Normalized GB dynamics probed at the band-edge for undoped and Mn-doped $CsPbI_3$ NCs at low excitation intensity with (d) 400 nm, (e) 500 nm, and (f) 570 nm excitation energy. Power loss as a function of the inverse carrier temperature for the exponential fitting data from Figure 4a-3c of undoped and Mn-doped $CsPbI_3$ NCs at low excitation intensity with (g) 400 nm, (h) 500 nm, and (i) 570 nm excitation energy.

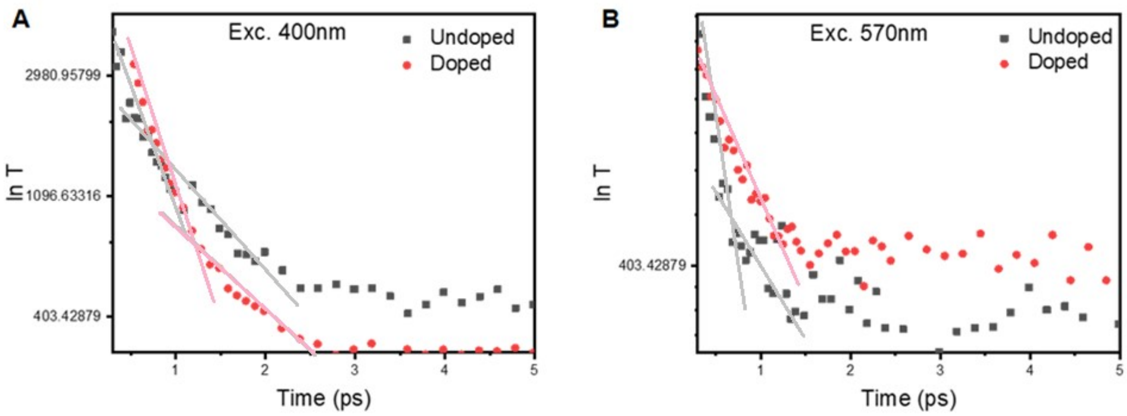

Figure S22. Time-dependent carrier temperature (ln T) for undoped and Mn-doped $CsPbI_3$ NCs at low excitation density (<N> < 1) with (a) 400 nm (3.1 eV) and (b) 570 nm (2.18 eV) excitation energy.

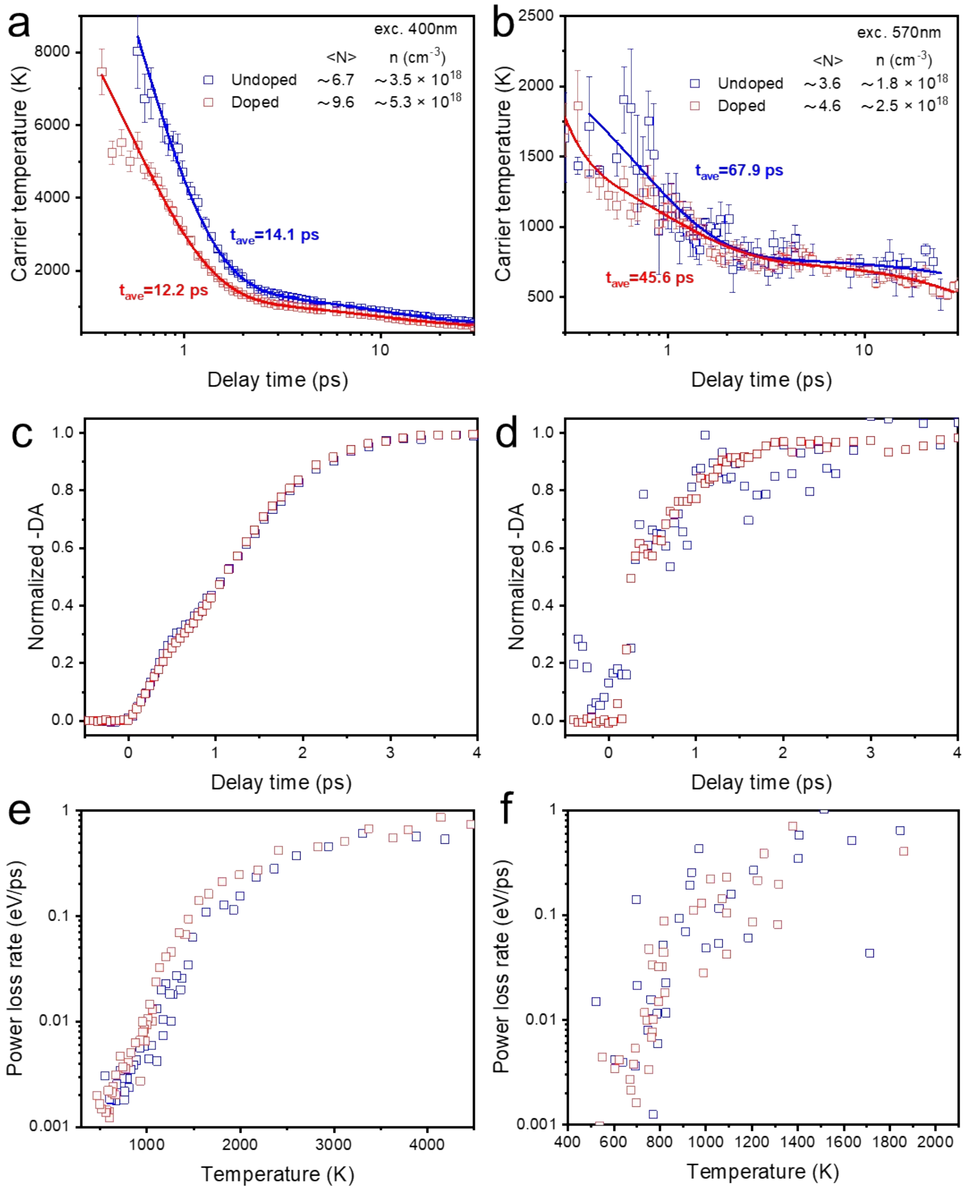

Figure S23. Time-dependent carrier temperature for undoped and Mn-doped $CsPbI_3$ NCs at high excitation density (<N> >> 1) with (a) 2.18 eV and (b) 3.1 eV excitation energy. Normalized GB dynamics probed at the band-edge for undoped and Mn-doped $CsPbI_3$ NCs at low excitation intensity with (c) 2.18 eV and (d) 3.1 eV excitation enrgy. Power loss as a function of the carrier temperature of Mn-doped $CsPbI_3$ NCs and corresponding exponential fitting at low excitation intensity with (e) 2.18 eV and (f) 3.1 eV excitation energy.

Table S1. The Absorption cross-sections of Mn doped $CsPbI_3$ at 400 nm, 500 nm and 570 nm. The values for 500 nm and 570 nm are calculated combining the absorption cross-section at 400 nm and absorption spectra in Figure 1c.

	0%	5%	7%	10%
400 nm (3.10 eV)	4.2×10^{-14} cm^2	6.0×10^{-14} cm^2	8.0×10^{-14} cm^2	1.4×10^{-14} cm^2
500 nm (2.48 eV)	1.5×10^{-14} cm^2	2.0×10^{-14} cm^2	2.8×10^{-14} cm^2	0.5×10^{-14} cm^2
570 nm (2.18 eV)	0.7×10^{-14} cm^2	0.9×10^{-14} cm^2	1.2×10^{-14} cm^2	0.2×10^{-14} cm^2

Table S2. Fit parameters for time-dependent carrier temperature of undoped $CsPbI_3$ NCs with 400 nm excitation at different intensities $n \approx 3.5 \times 10^{17}$ (I_1), 1.7×10^{18} (I_2), 3.5×10^{18} (I_3) and 6.9×10^{18} (I_4) photon/cm^2/pulse, corresponding to $\langle N \rangle \approx 0.7$, 3.7 6.7 and 13.5, respectively. The unit of time t is *ps*.

	A_1	t_1	A_2	t_2	A_3	t_3	t_{ave}
I_1	0.77	0.2	0.22	0.7	0.02	19.2	9.9
I_2	0.74	0.7	0.21	0.7	0.05	24.0	16.4
I_3	0.60	0.6	0.35	0.6	0.06	20.8	14.1
I_4	0.56	0.9	0.32	0.9	0.13	5.4	3.0

Table S3. Fit parameters for time-dependent carrier temperature of Mn doped $CsPbI_3$ NCs with 400 nm excitation at different intensities $n \approx 5.3 \times 10^{17}$ (I_1), 2.7×10^{18} (I_2), 5.3×10^{18} (I_3) and 1.1×10^{19} (I_4) photon/cm^2/pulse, corresponding to $\langle N \rangle \approx$ 1.0, 4.8 9.6 and 19.2, respectively. The unit of time t is *ps*.

	A_1	t_1	A_2	t_2	A_3	t_3	t_{ave}
I_1	0.97	0.1	0.03	0.4	0.0005	29.4	3.8
I_2	0.97	0.7	0.03	14.0	-	-	8.3
I_3	0.93	0.6	0.07	17.7	-	-	12.2
I_4	0.89	0.8	0.11	12.4	-	-	8.2

Table S4. Fit parameters for time-dependent carrier temperature of undoped $CsPbI_3$ NCs with 500 nm excitation at different intensities $n \approx 3.0 \times 10^{17}$ (I_1), 1.5×10^{18} (I_2), 3.0×10^{18} (I_3) and 4.4×10^{19} (I_4) photon/cm^2/pulse, corresponding to $\langle N \rangle \approx$ 0.6, 2.9 5.8 and 8.7, respectively. The unit of time *t* is *ps*.

	A_1	t_1	A_2	t_2	A_3	t_3	t_{ave}
I_1	0.94	0.4	0.06	4.1	-	-	1.8
I_2	0.89	0.5	0.11	27.4	-	-	23.7
I_3	0.84	0.6	0.16	23.2	-	-	20.2
I_4	0.87	0.7	0.13	12.7	-	-	9.7

Table S5. Fit parameters for time-dependent carrier temperature of Mn doped $CsPbI_3$ NCs with 500 nm excitation at different intensities $n \approx 4.2 \times 10^{17}$ (I_1), 2.1×10^{18} (I_2), 4.2×10^{18} (I_3) and 6.4×10^{18} (I_4) photon/cm^2/pulse, corresponding to $\langle N \rangle \approx 0.8$, 3.9 7.7 and 11.5, respectively. The unit of time *t* is *ps*.

	A_1	t_1	A_2	t_2	A_3	t_3	t_{ave}
I_1	1	0.6	-	-	-	-	0.6
I_2	0.93	0.6	0.07	18.8	-	-	13.1
I_3	0.86	0.7	0.14	21.4	-	-	17.9
I_4	0.86	0.6	0.14	11.7	-	-	9.0

Table S6. Fit parameters for time-dependent carrier temperature of undoped $CsPbI_3$ NCs with 570 nm excitation at different intensities $n \approx 2.6 \times 10^{17}$ (I_1), 7.9×10^{17} (I_2), 1.8×10^{18} (I_3) and 2.9×10^{18} (I_4) photon/cm^2/pulse, corresponding to $\langle N \rangle \approx 0.5$, 1.5, 3.6 and 5.6, respectively. The unit of time *t* is *ps*.

	A_1	t_1	A_2	t_2	A_3	t_3	t_{ave}
I_1	0.95	0.1	0.05	0.7	-	-	0.3
I_2	1	0.5	-	-	-	-	0.5
I_3	0.82	0.7	0.18	71	-	-	67.9
I_4	0.87	0.5	0.13	16.8	-	-	14.1

Table S7. Fit parameters for time-dependent carrier temperature of Mn doped $CsPbI_3$ NCs with 570 nm excitation at different intensities $n \approx 3.6 \times 10^{17}$ (I_1), 1.1×10^{18} (I_2), 2.5×10^{18} (I_3) and 4.0

× 10^{18} (I_4) photon/cm^2/pulse, corresponding to ⟨*N*⟩ ≈ 0.7, 2.0, 4.6 and 7.2, respectively. The unit of time *t* is *ps*.

	A_1	t_1	A_2	t_2	A_3	t_3	t_{ave}
I_1	1	0.4	-	-	-	-	0.4
I_2	1	0.6	-	-	-	-	0.6
I_3	0.92	0.1	0.05	0.9	0.03	49.4	45.6
I_4	0.81	0.7	0.19	12.1	-	-	9.8

Table S8. Fit parameters for time-dependent carrier temperature of Mn doped $CsPbI_3$ NCs with 400 nm excitation at different doping concentration. $n \approx 3.5 \times 10^{17}$ (0%), 5.3×10^{17} (5%), 8.0×10^{17} (7%) and 2.9×10^{17} (10%) photon/cm^2/pulse, corresponding to ⟨*N*⟩ ≈ 0.7, 1.0, 1.2 and 0.2, respectively. The unit of time *t* is *ps*.

	A_1	t_1	A_2	t_2	A_3	t_3	t_{ave}
0%	0.85	0.2	0.15	0.9	-	-	0.5
5%	0.97	0.1	0.03	0.5	-	-	0.2
7%	0.91	0.1	0.09	0.4	-	-	0.2
10%	0.97	0.3	0.03	1.4	-	-	0.5

Table S9. Fit parameters for time-dependent carrier temperature of Mn doped $CsPbI_3$ NCs with 500 nm excitation at different doping concentration. $n \approx 3.0 \times 10^{17}$ (0%), 4.2×10^{17} (5%), 6.7×10^{17}

(7%) and 2.5 × 10^{17} (10%) photon/cm^2/pulse, corresponding to ⟨*N*⟩ ≈ 0.6, 0.8, 1.1 and 0.2, respectively. The unit of time *t* is *ps*.

	A_1	t_1	A_2	t_2	A_3	t_3	t_{ave}
0%	0.94	0.1	0.06	4.1	-	-	1.8
5%	1	0.6	-	-	-	-	0.6
7%	1	0.4	-	-	-	-	0.4
10%	0.95	0.1	0.03	0.2	0.02	0.5	0.1

Table S10. Fit parameters for time-dependent carrier temperature of Mn doped $CsPbI_3$ NCs with 570 nm excitation at different doping concentration. *n* ≈ 2.6 × 10^{17} (0%), 3.6 × 10^{17} (5%), 5.5 × 10^{17} (7%) and 1.9 × 10^{17} (10%) photon/cm^2/pulse, corresponding to ⟨*N*⟩ ≈ 0.5, 0.7, 0.9 and 0.1, respectively. The unit of time *t* is *ps*.

	A_1	t_1	A_2	t_2	A_3	t_3	t_{ave}
0%	0.95	0.1	0.05	0.7	-	-	0.3
5%	1	0.4	-	-	-	-	0.4
7%	1	0.6	-	-	-	-	0.6
10%	0.55	0.3	0.42	0.4	0.03	2.0	0.6

Table S11. Fit parameters of Figure S21 for carrier temperature decay kinetics of Mn-doped $CsPbI_3$ NCs ($N \leq 1$) with 400 nm and 570 nm excitation at low intensity ($N \leq 1$). The unit of time *t* is *ps*.

**Exc. Wavelength**		**A_1**	**t_1**	**A_2**	**t_2**	**t_{ave}**
**400 nm**	Undoped	0.85	0.2	0.15	0.9	0.5
	Doped	0.97	0.1	0.03	0.5	0.2
**500 nm**	Undoped	0.94	0.1	0.06	4.1	1.8
	Doped	1	0.6	-	-	0.6
**570 nm**	Undoped	0.95	0.1	0.05	0.7	0.3
	Doped	1	0.4	-	-	0.4

References

(1) Perdew, J. P.; Burke, K.; Ernzerhof, M. Generalized Gradient Approximation Made Simple. *Phys. Rev. Lett.* **1996**, *77*, 3865–3868.

(2) Kresse, G.; Hafner, J. Ab Initio Molecular Dynamics for Liquid Metals. *Phys. Rev. B* **1993**, *47*, 558–561.

(3) Blöchl, P. E. Projector Augmented-Wave Method. *Phys. Rev. B* **1994**, *50*, 17953–17979.

(4) Welter, E.; Chernikov, R.; Herrmann, M.; Nemausat, R. A Beamline for Bulk Sample X-Ray Absorption Spectroscopy at the High Brilliance Storage Ring PETRA III. *AIP Conf. Proc.* **2019**, *2054*, 1–6.
